# Supplementary figures and images for: Ecological niche modeling as an effective tool to predict the distribution of freshwater organisms: The case of the Sabaleta Brycon henni (Eigenmann, 1913)
Source: PLoS One. 2021 Mar 3;16(3):e0247876. doi: 10.1371/journal.pone.0247876 (PMC7928524; doi:10.1371/journal.pone.0247876)

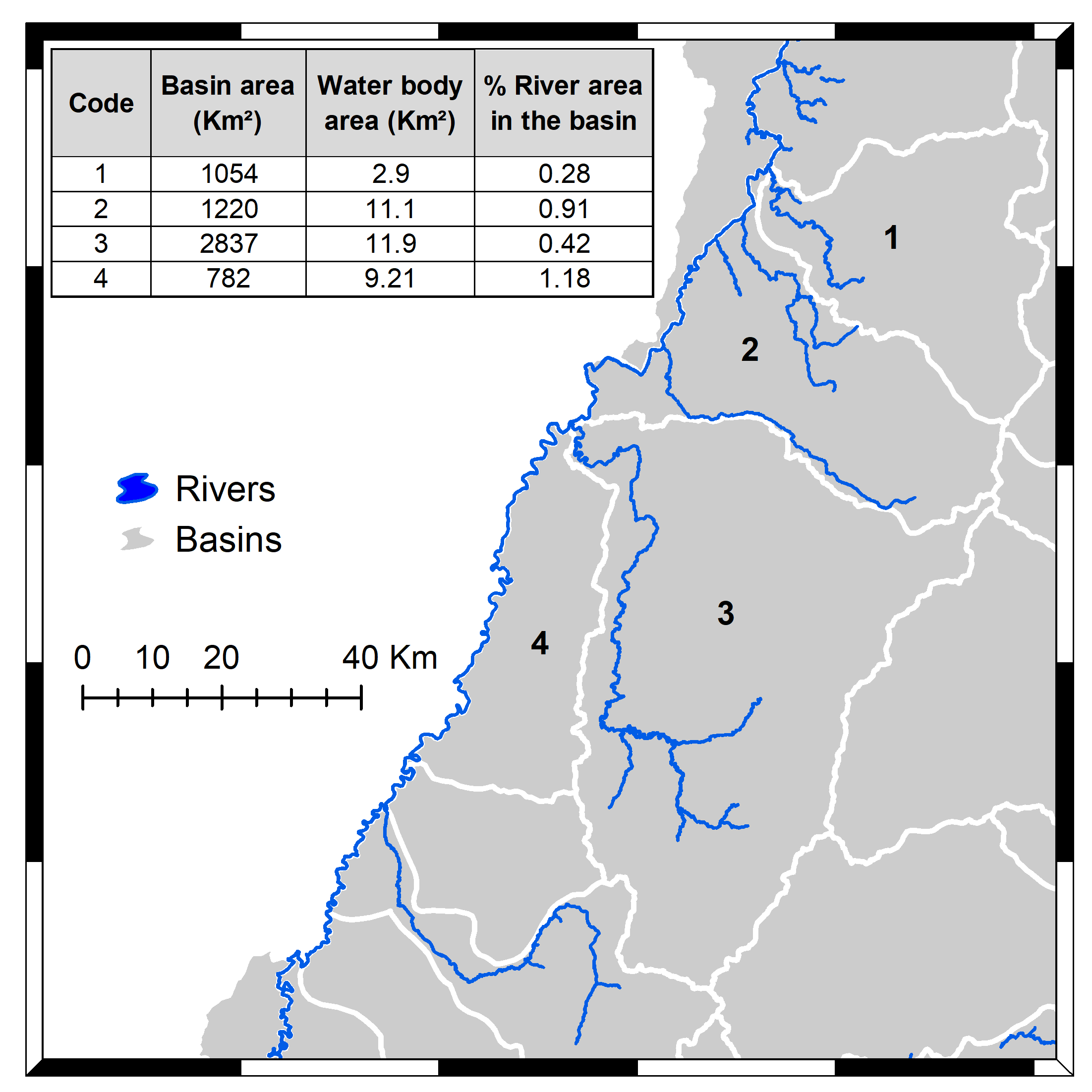

Supplement: S1 Fig — The shapefile of hydrographic basins and rivers of Colombia was obtained from IGAC (https://geoportal.igac.gov.co). All other products were produced by the authors and are copyright-free. (TIF) [file pone.0247876.s001.tif]

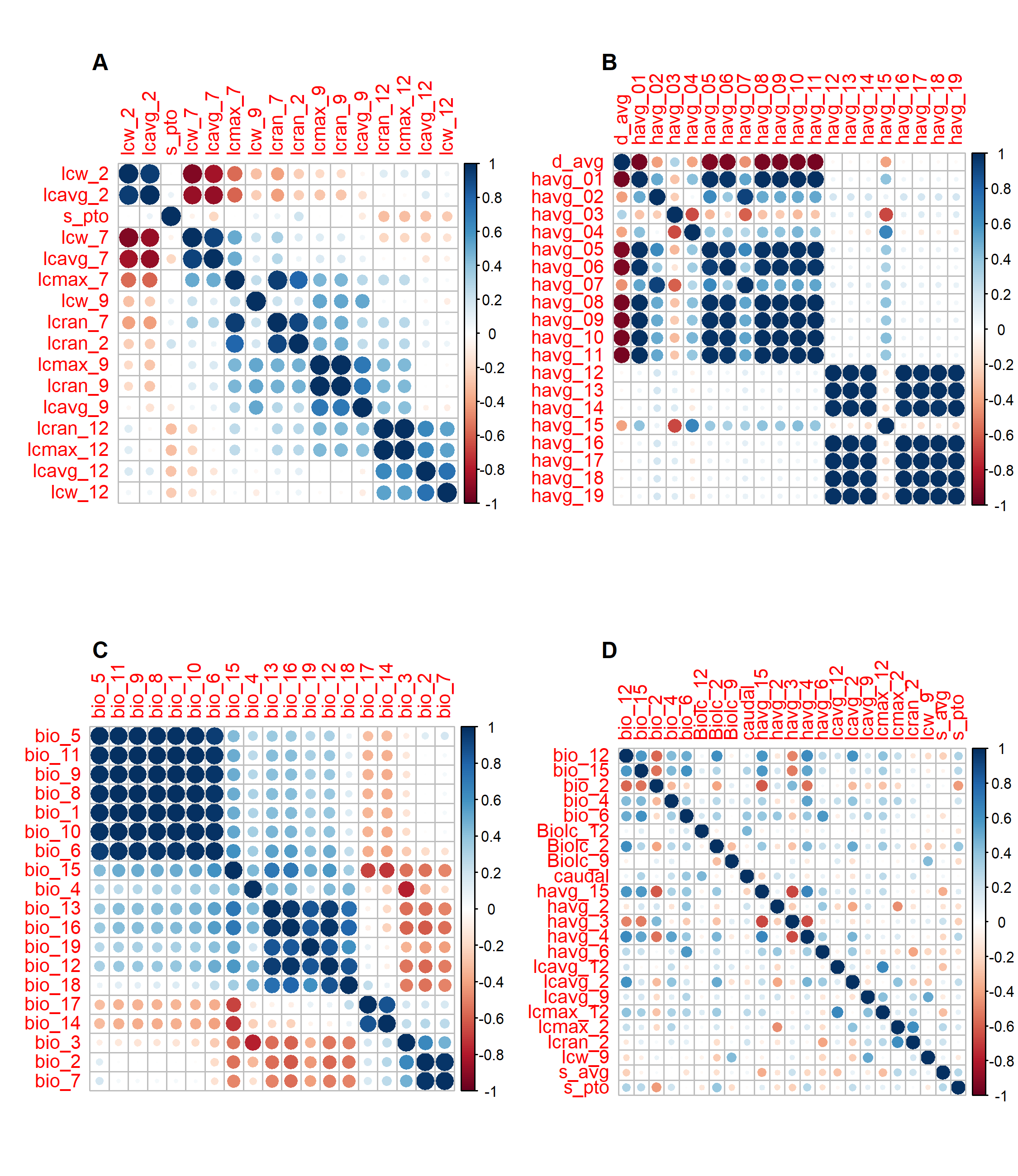

Supplement: S2 Fig — (A) Land use, (B) upstream climatic variables (average temperature and precipitation throughout the basin), (C) climatic data points and (D) set of variables that we recognized as most relevant for the species with no correlation (<0.75). For each predictor information, see S2 Table. (TIF) [file pone.0247876.s002.tif]

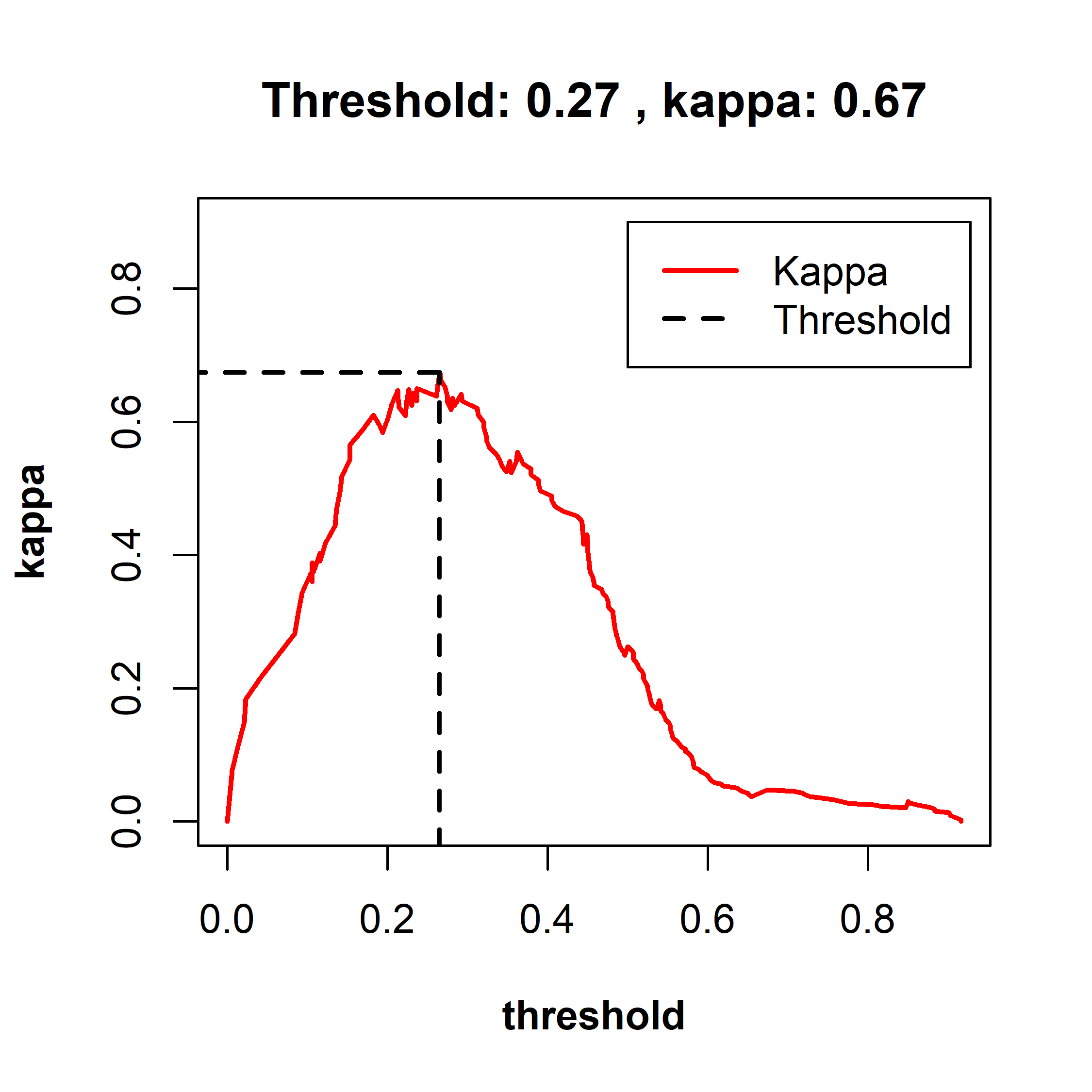

Supplement: S3 Fig — Kappa defines the precision of the prediction in relation to that expected by chance, it corresponds to the proportion of all test records that indicate agreement between the classifier and the observations. (TIF) [file pone.0247876.s003.tif]

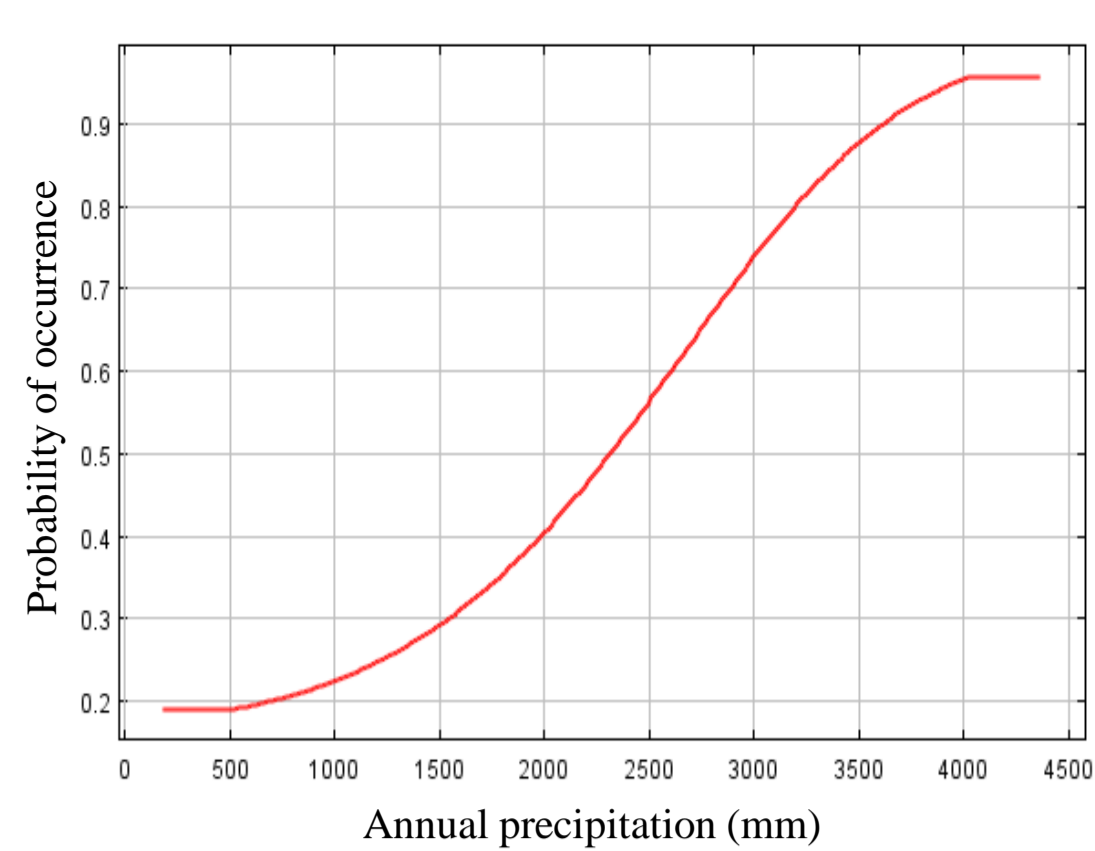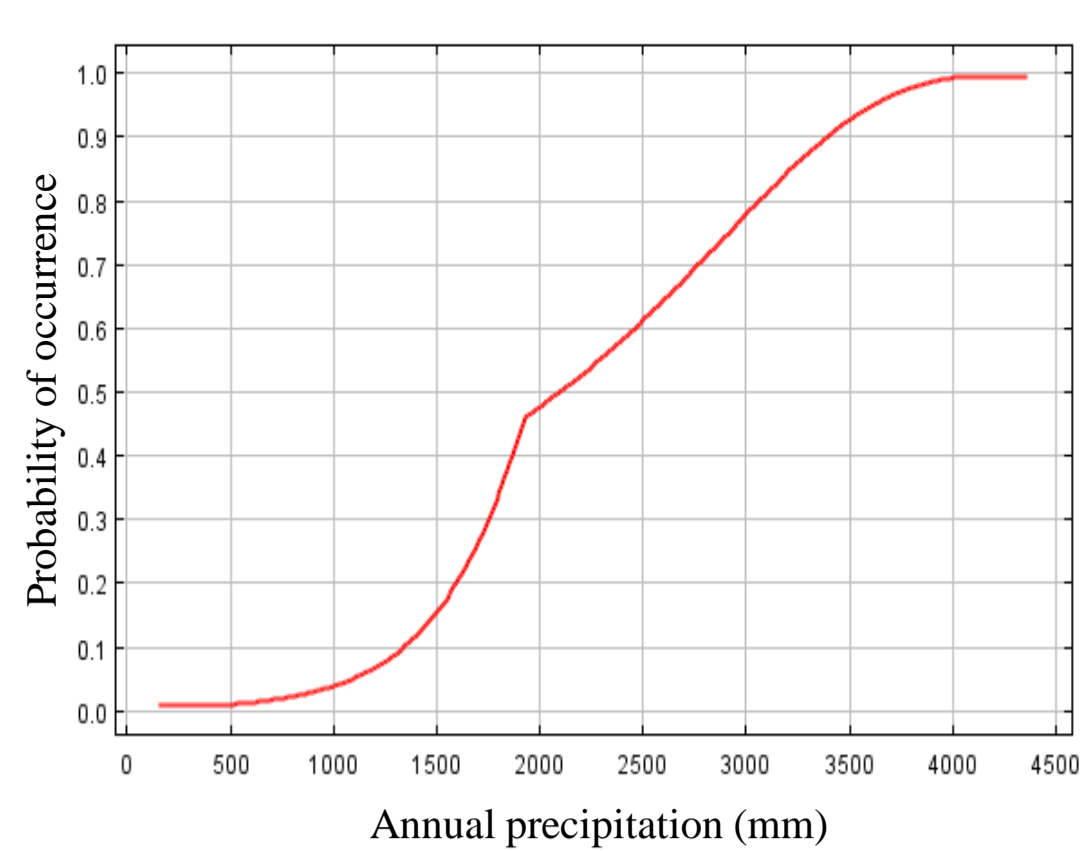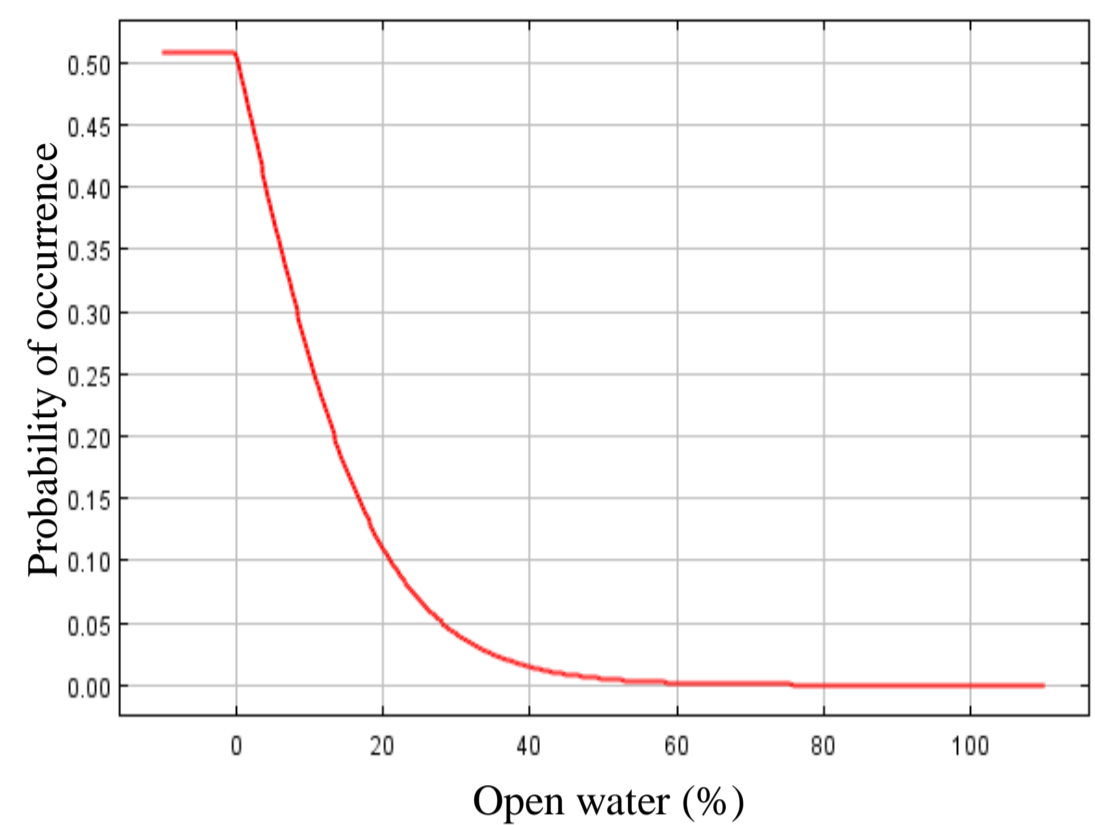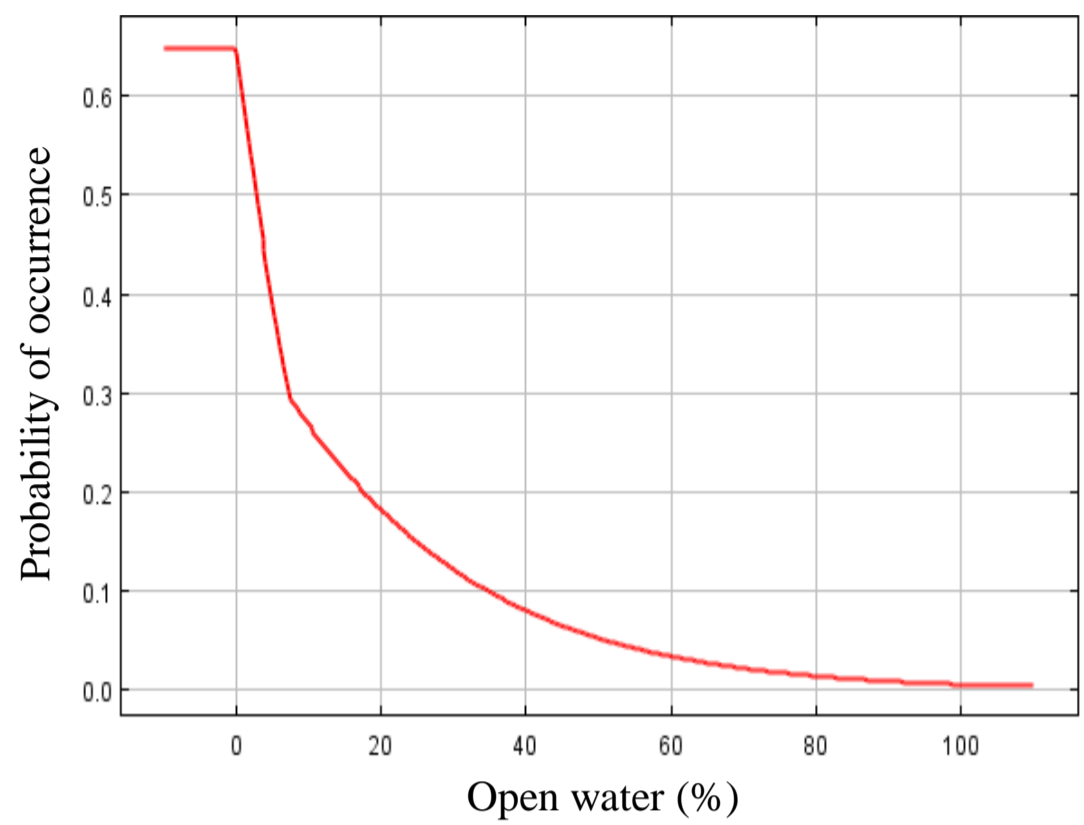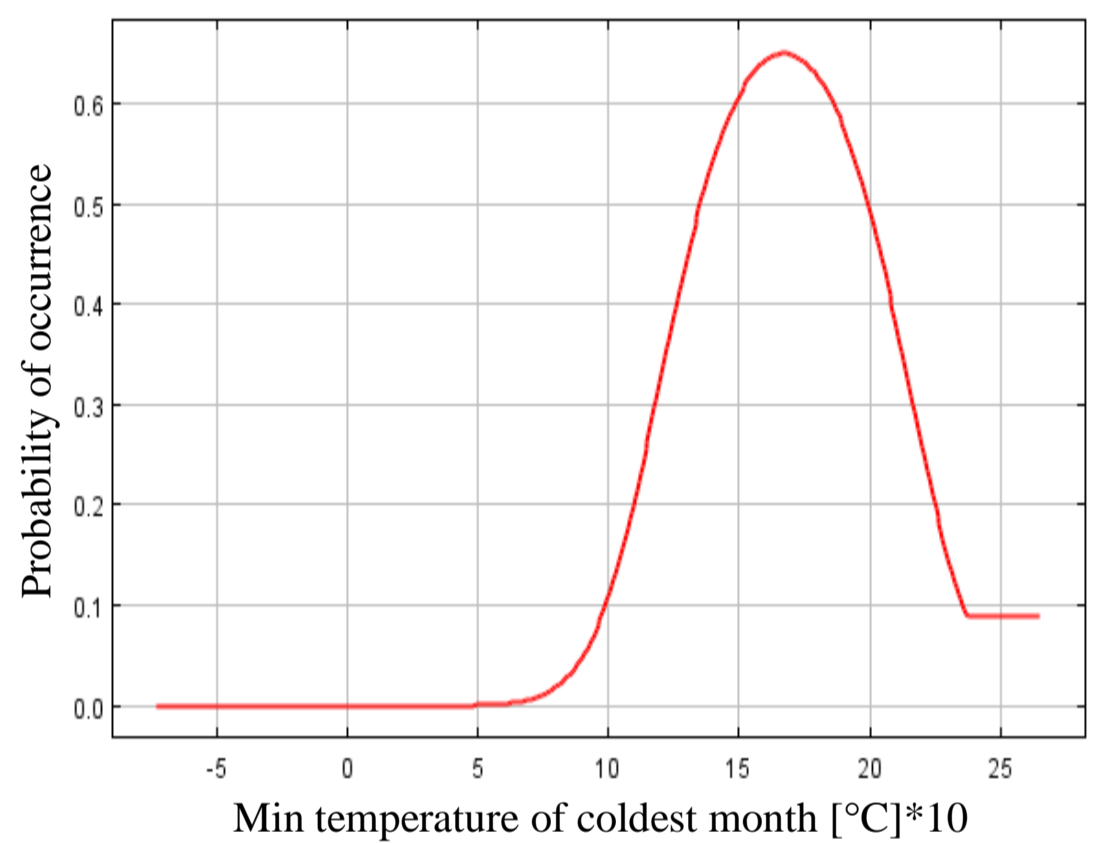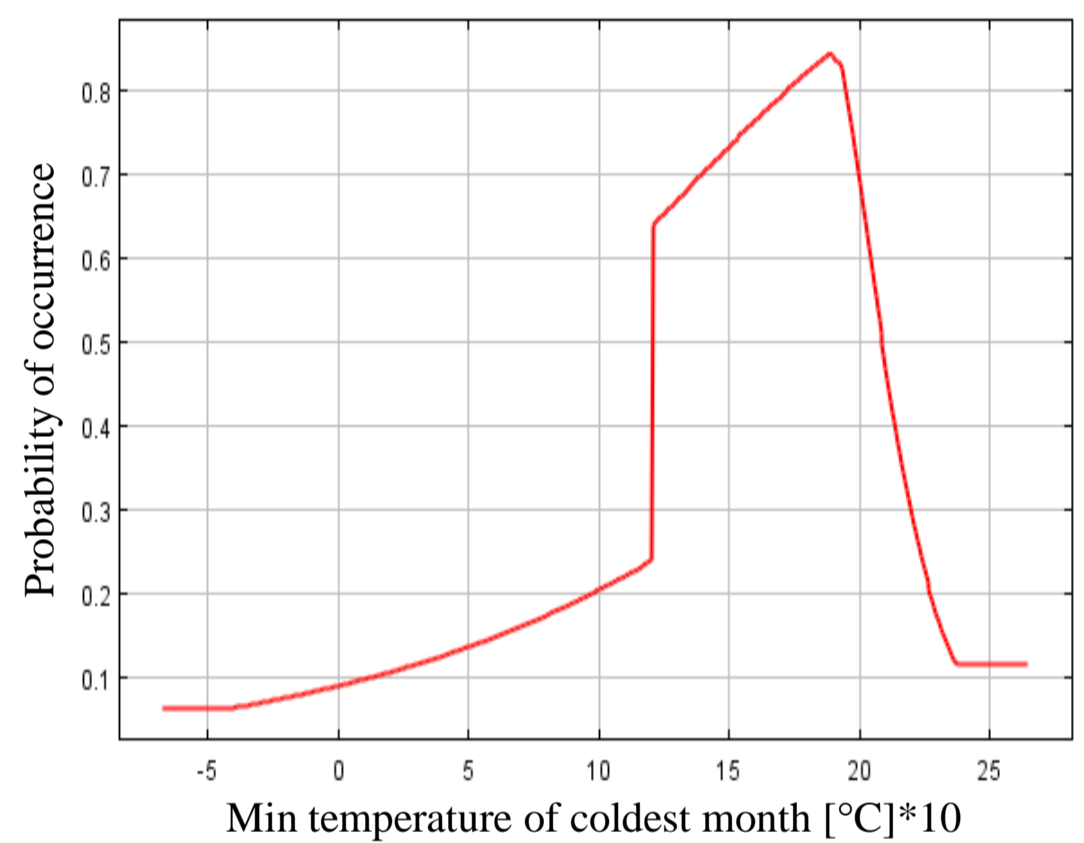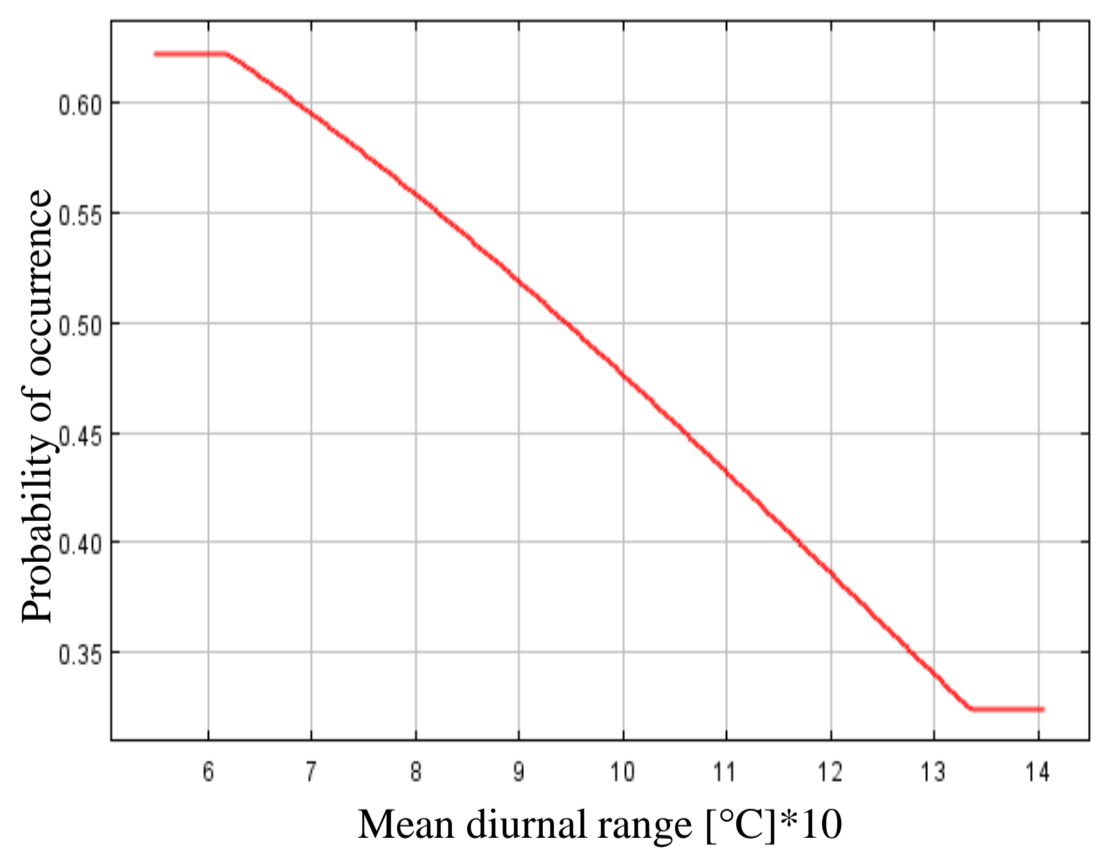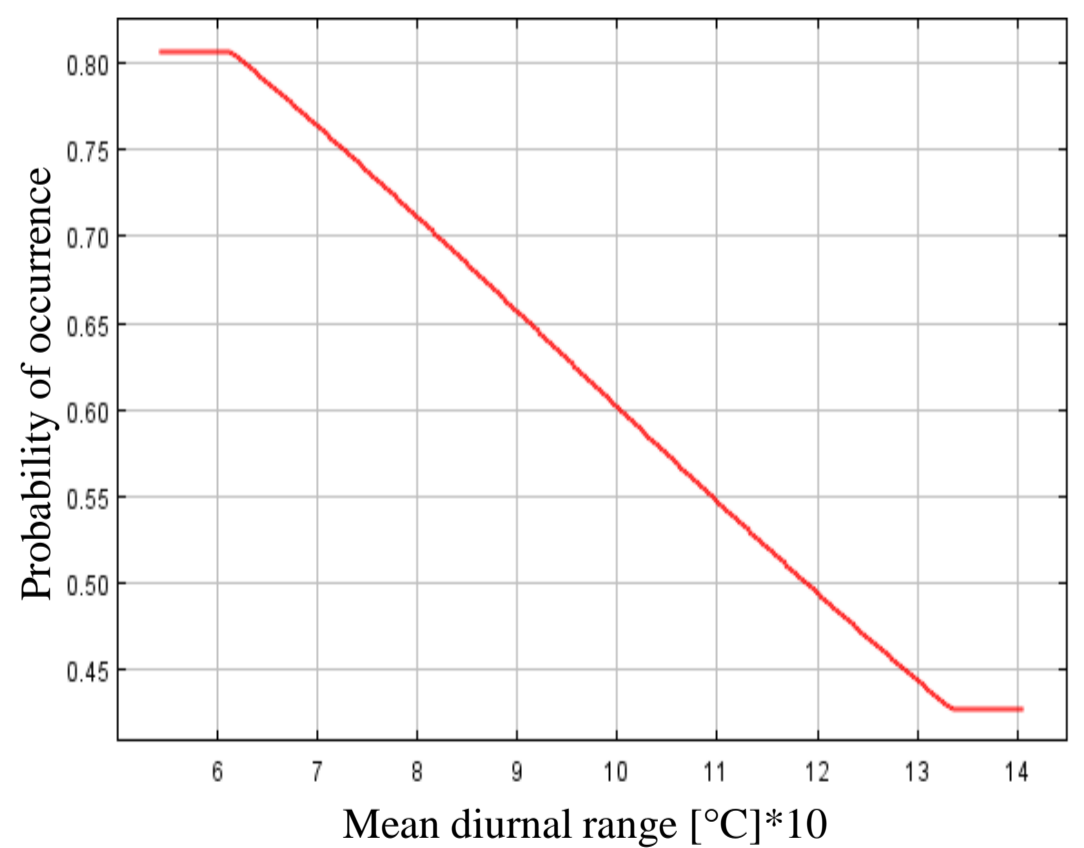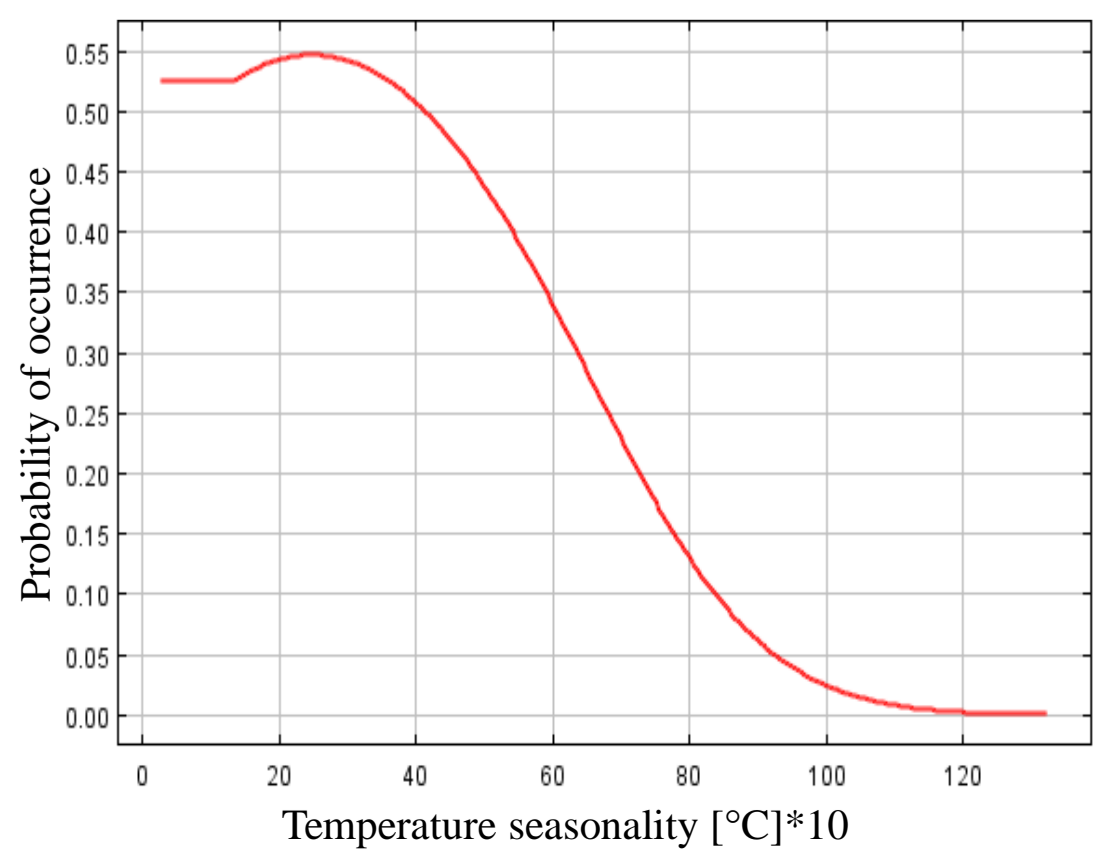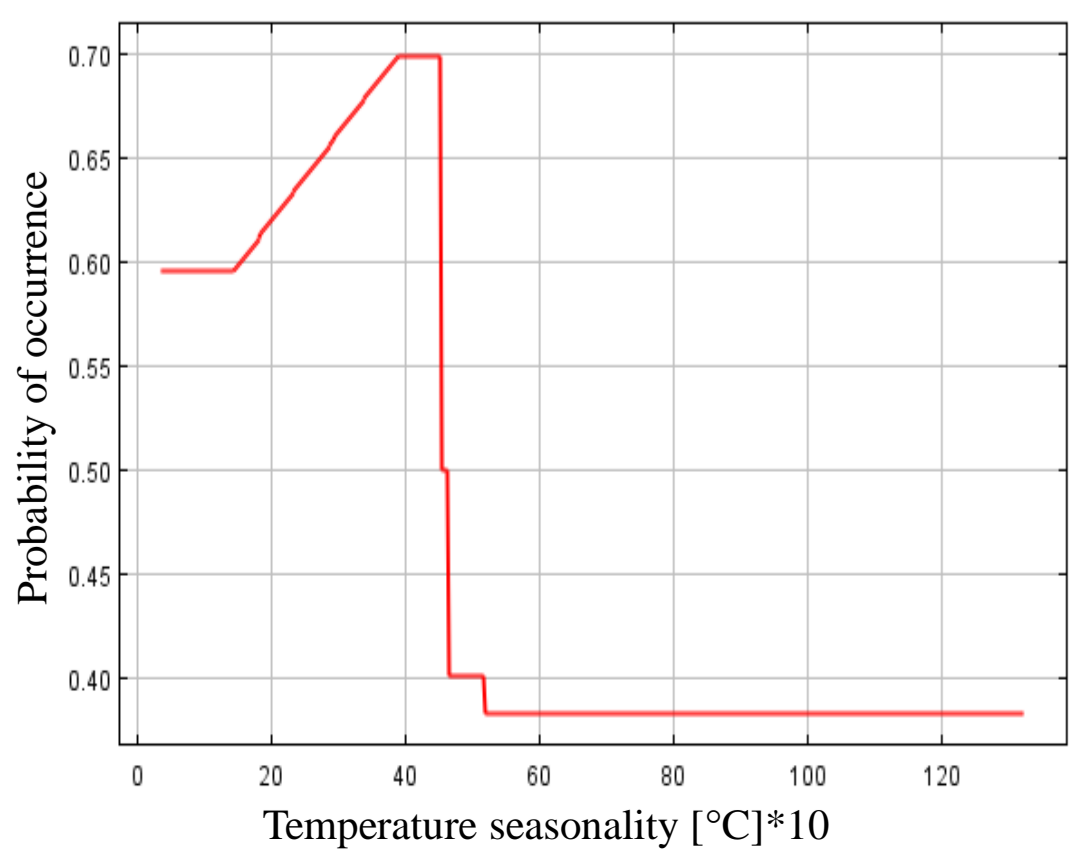

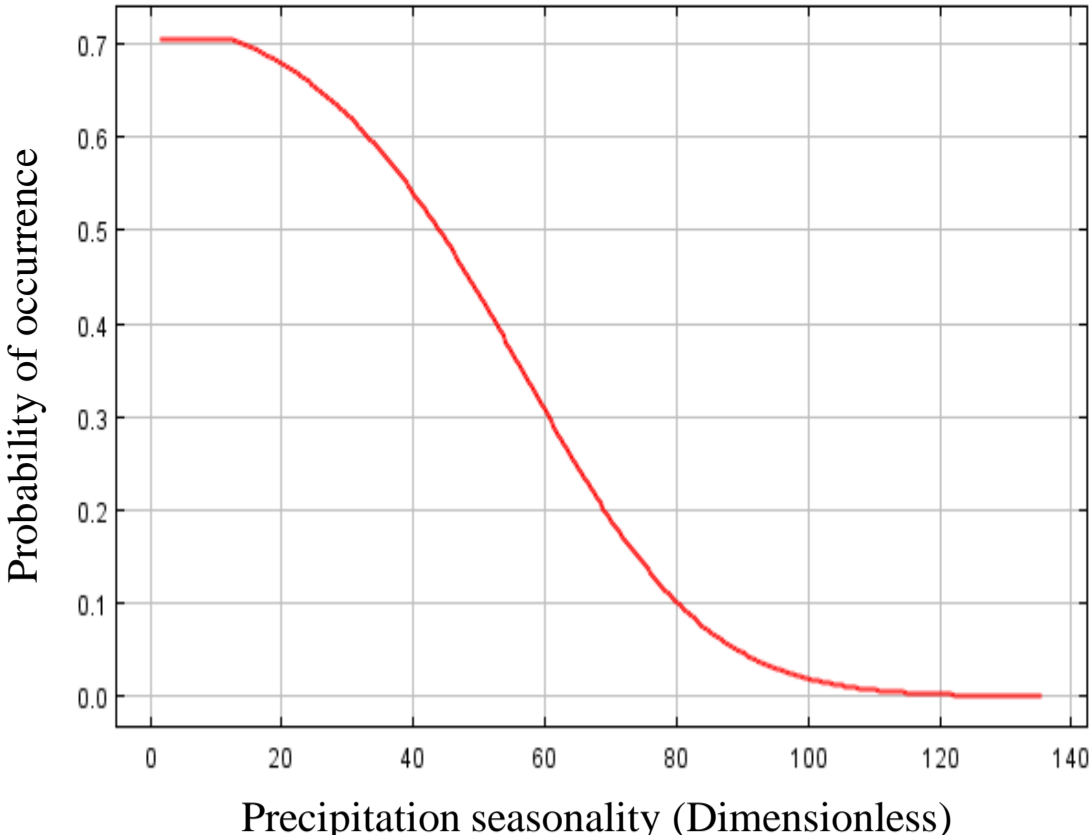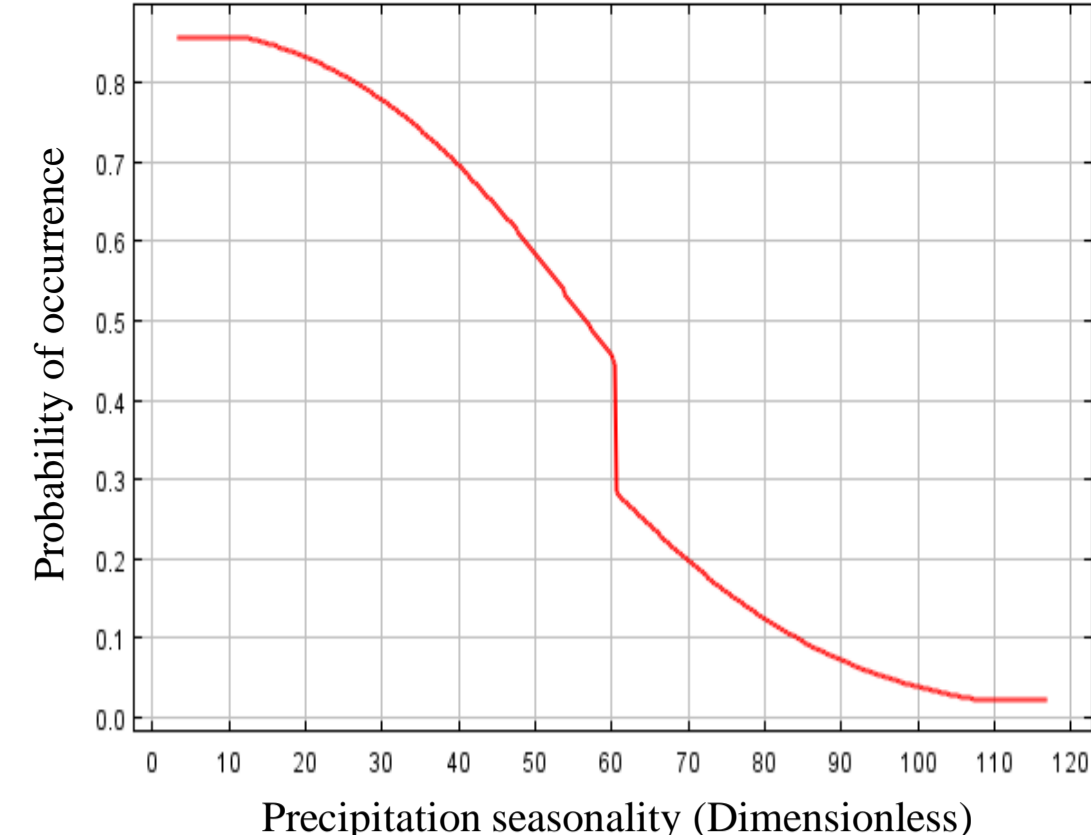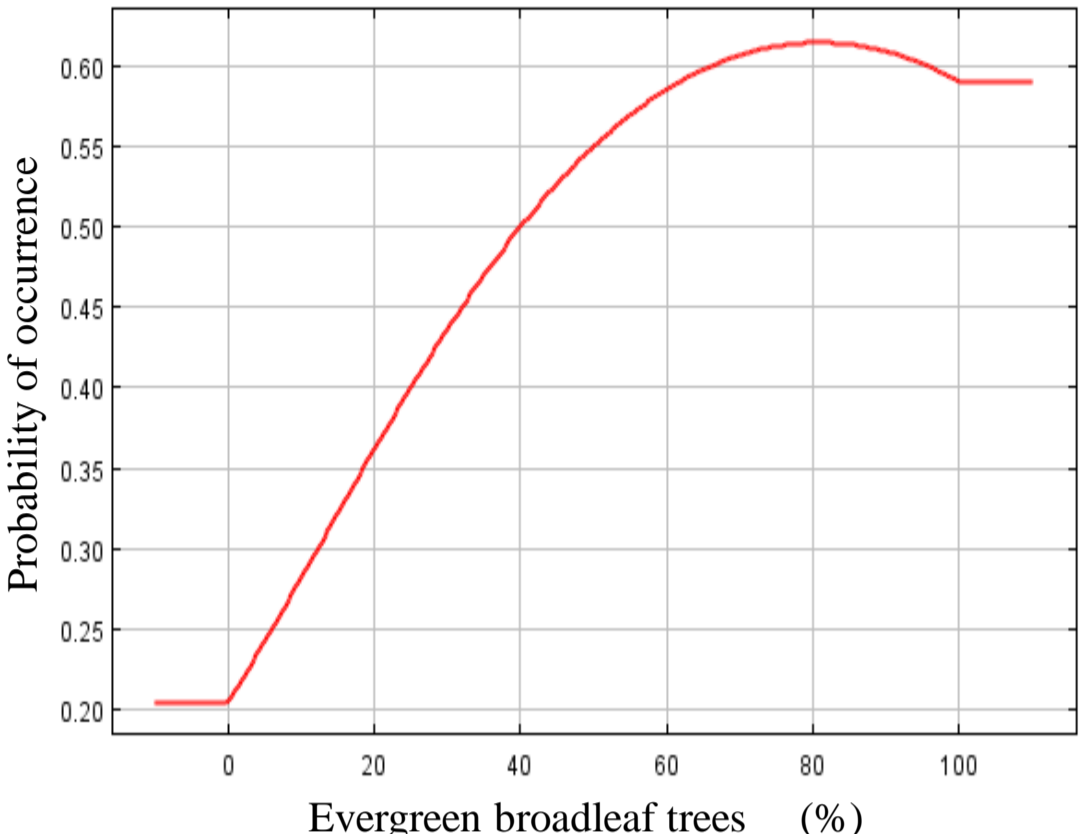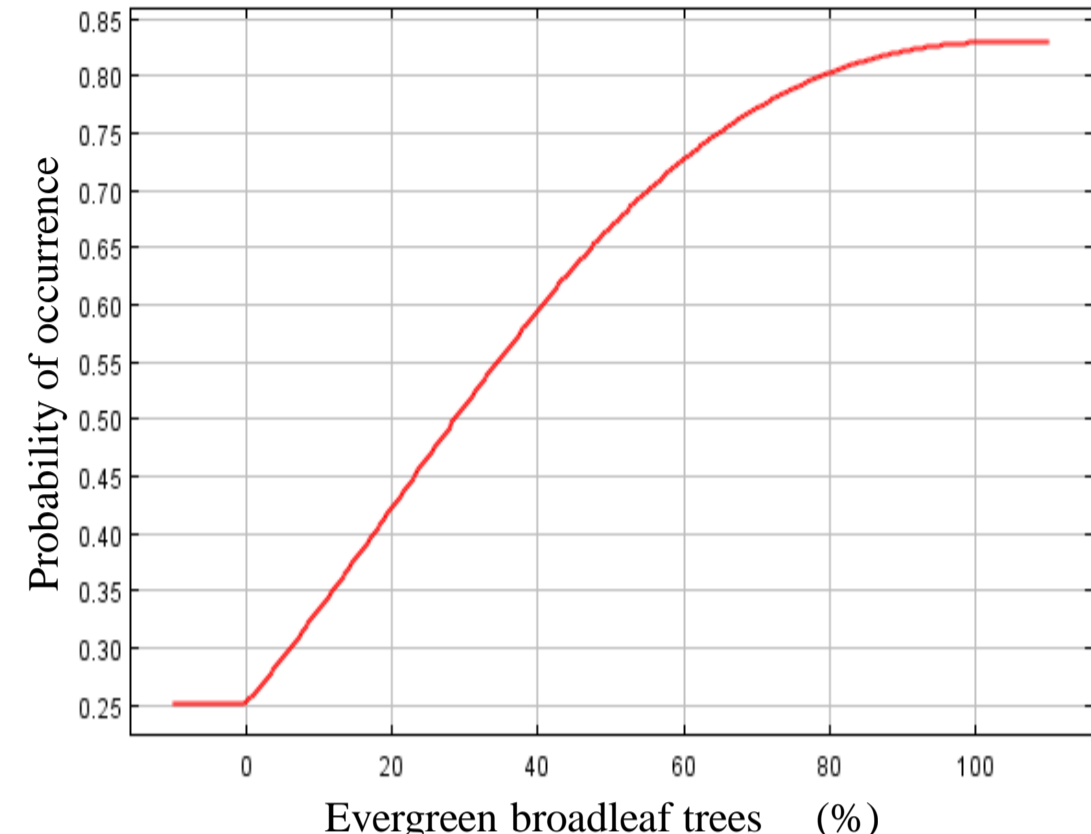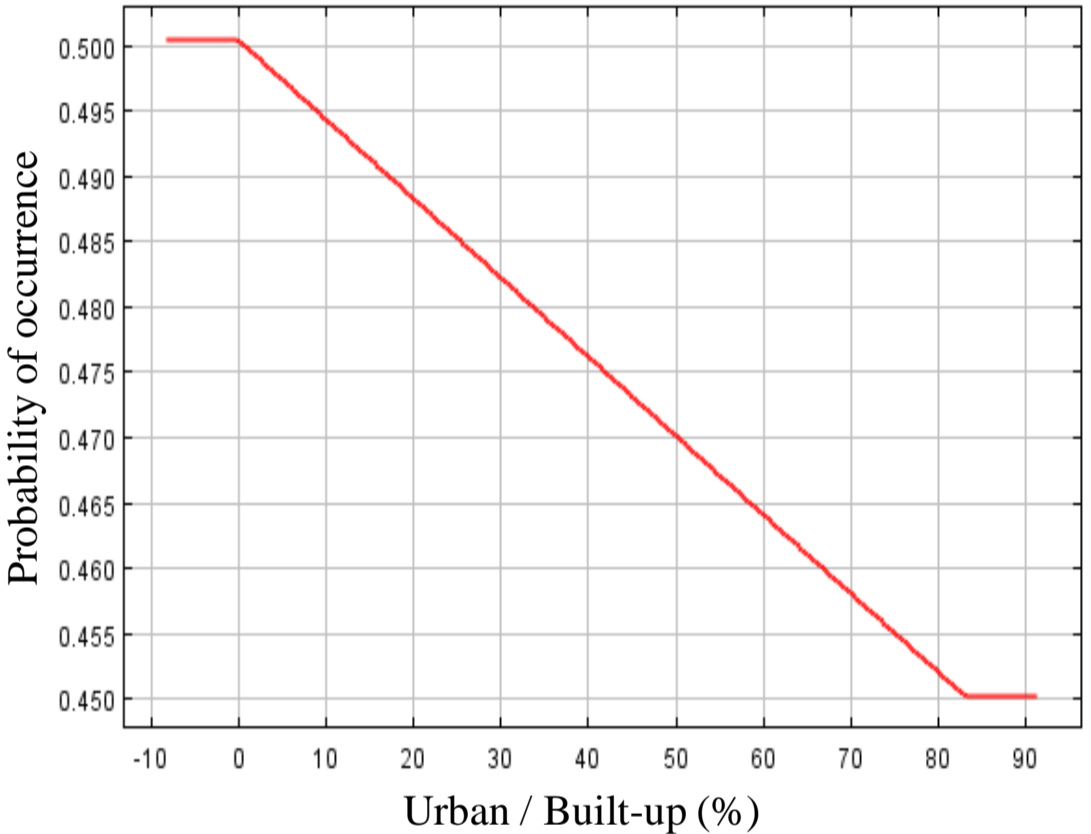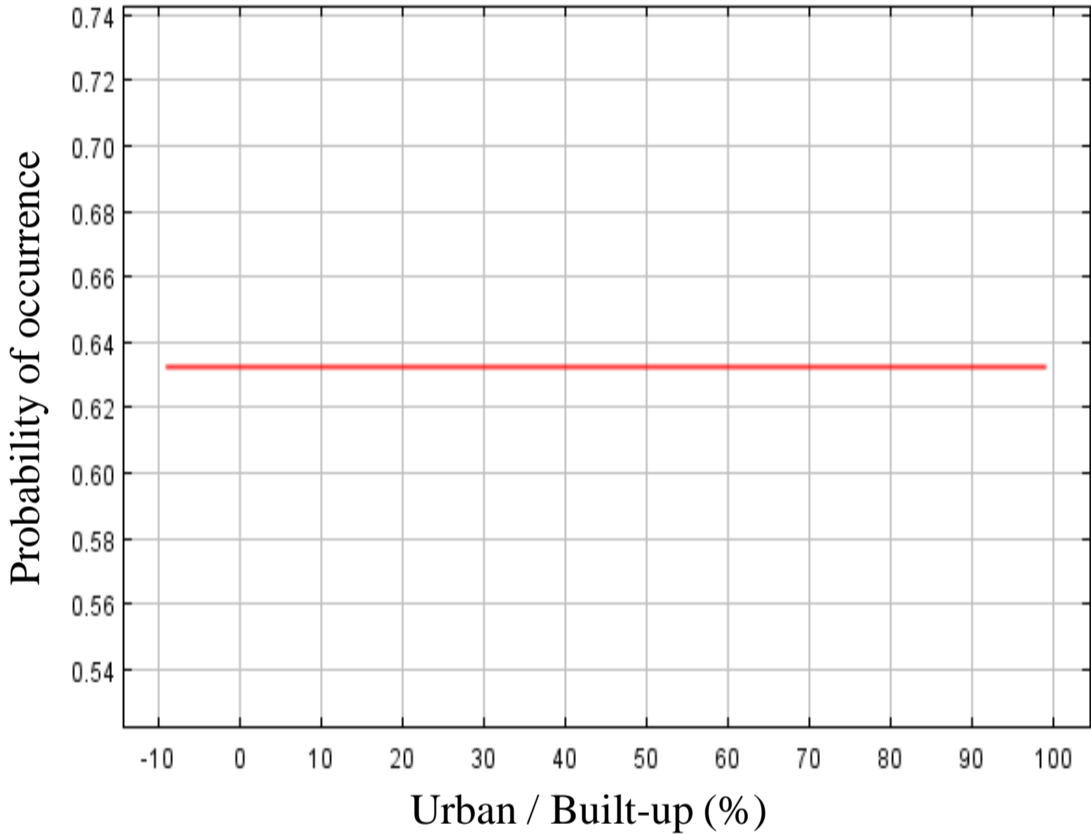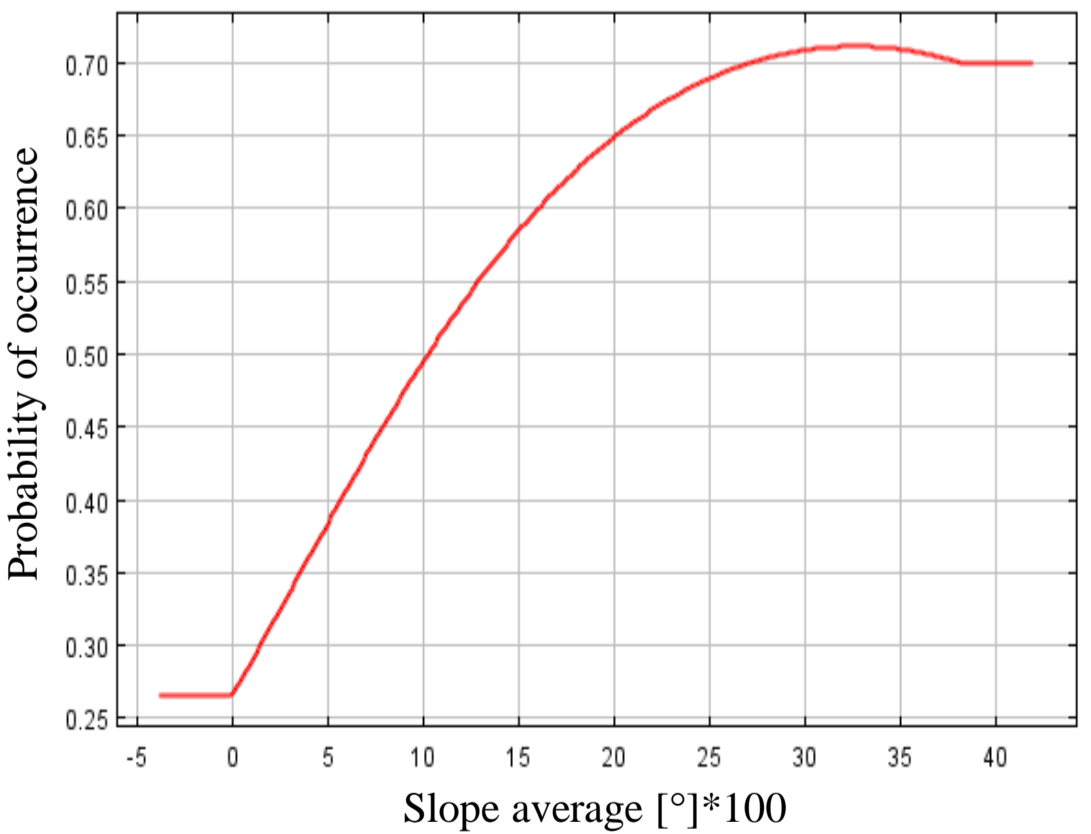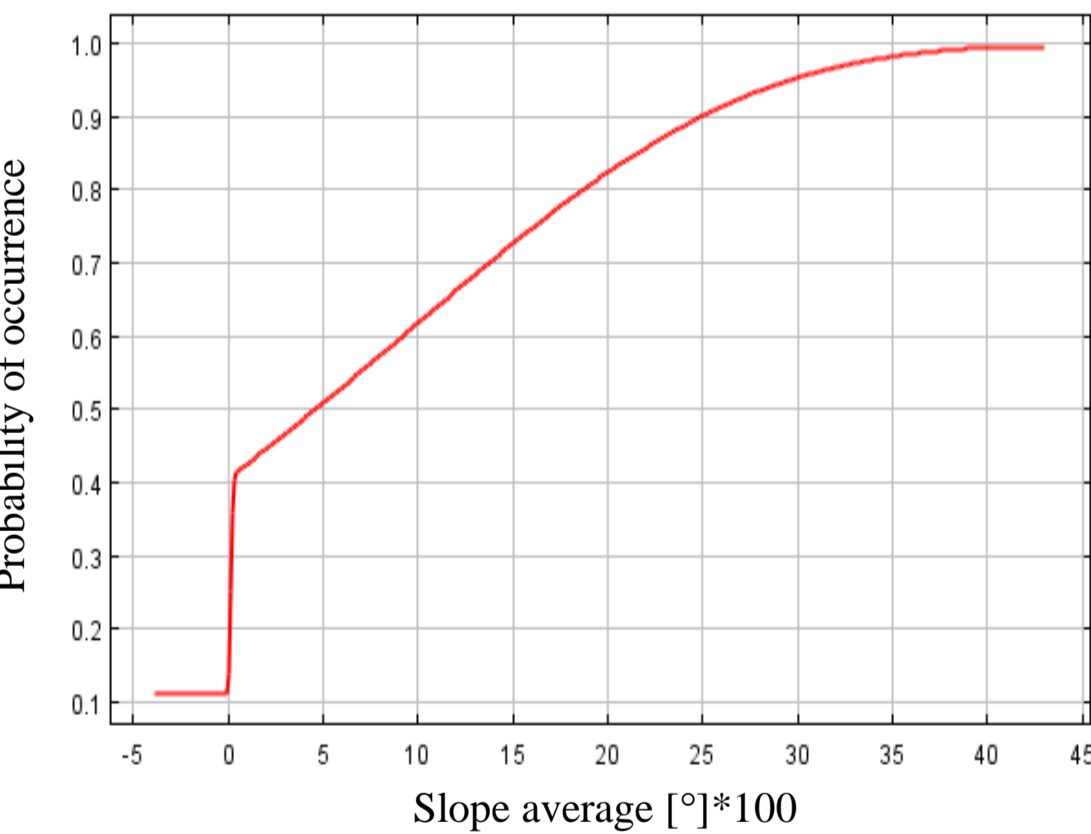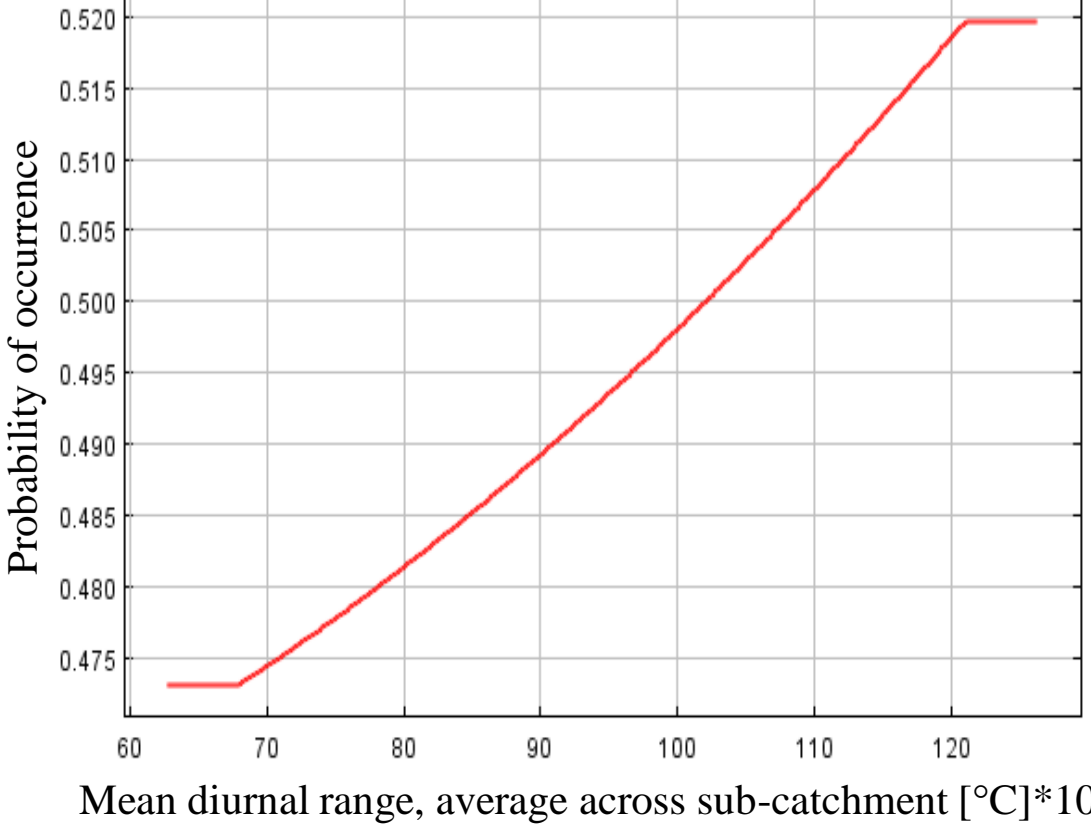

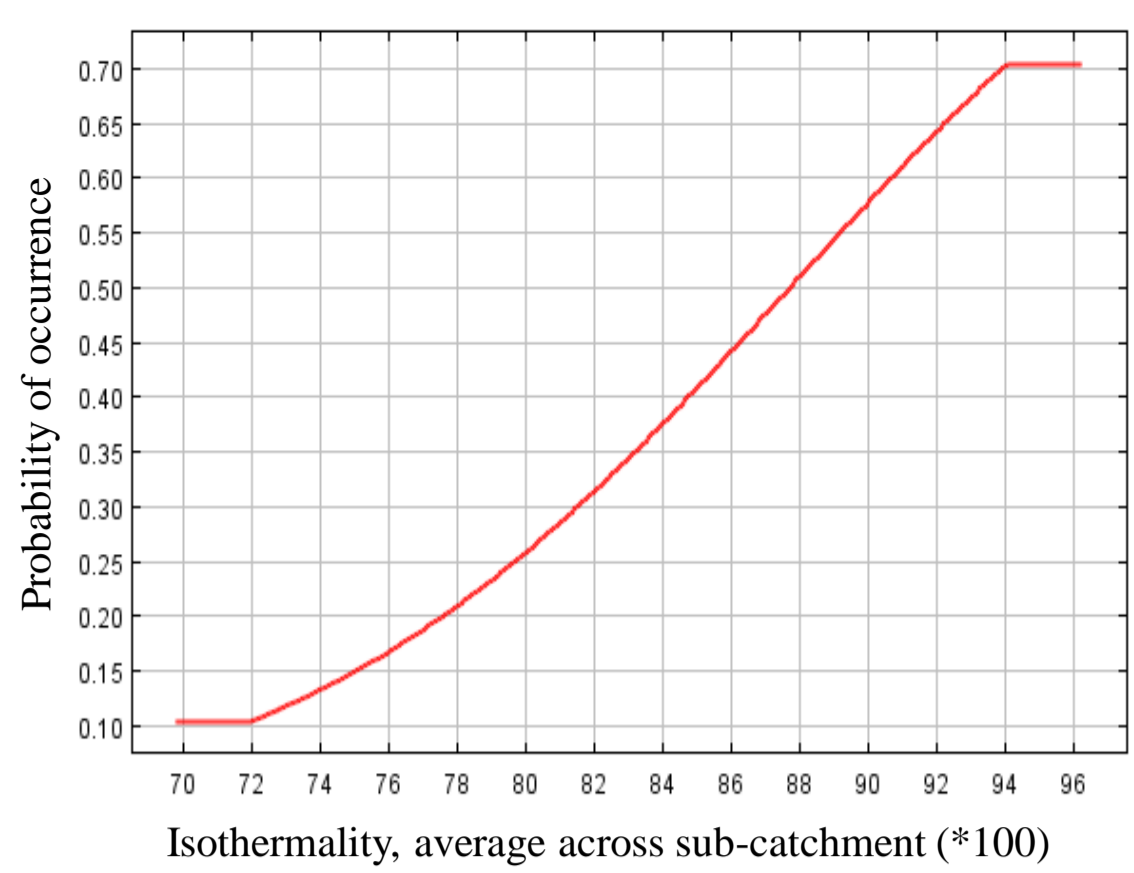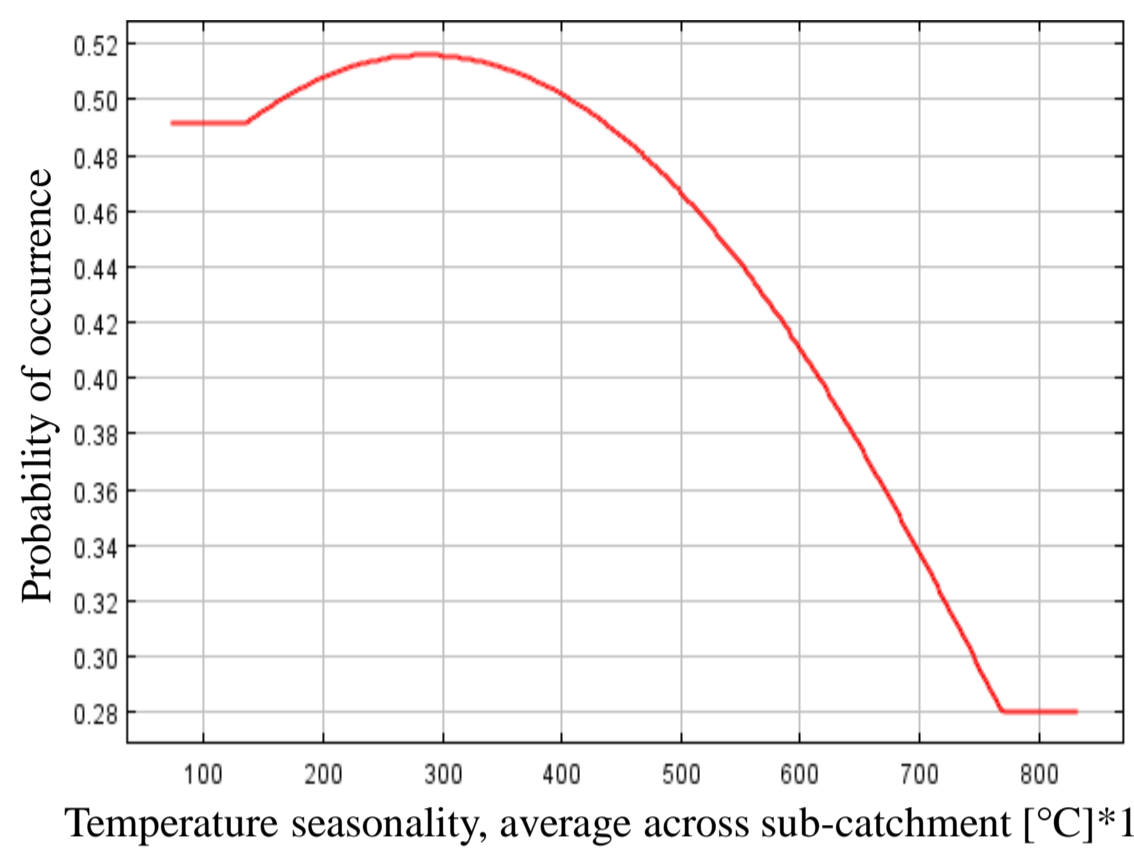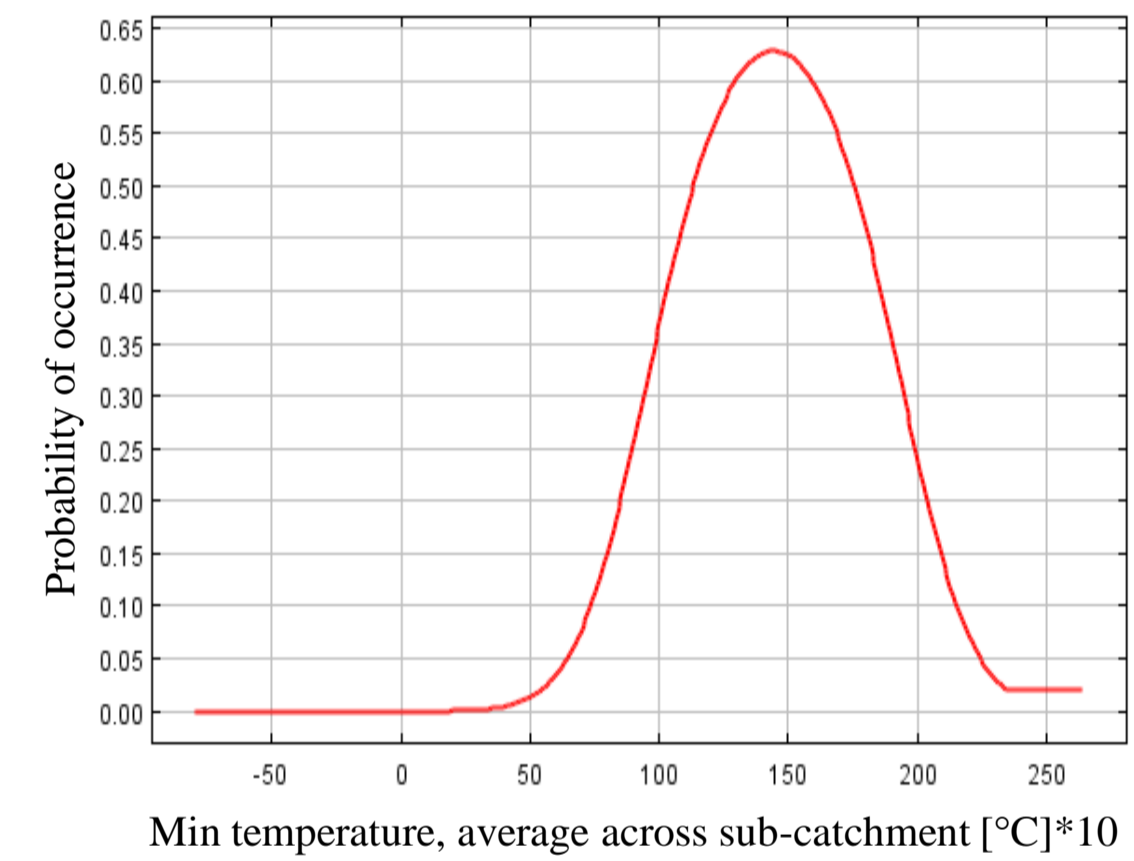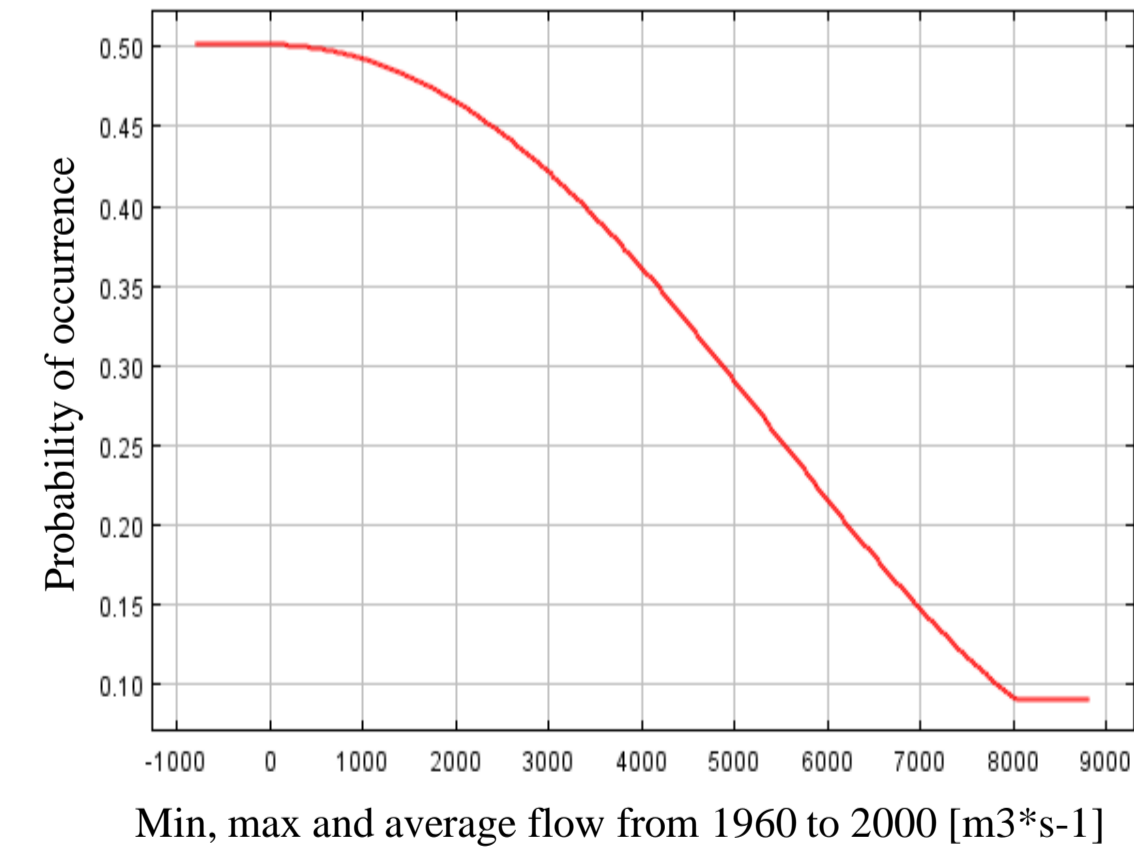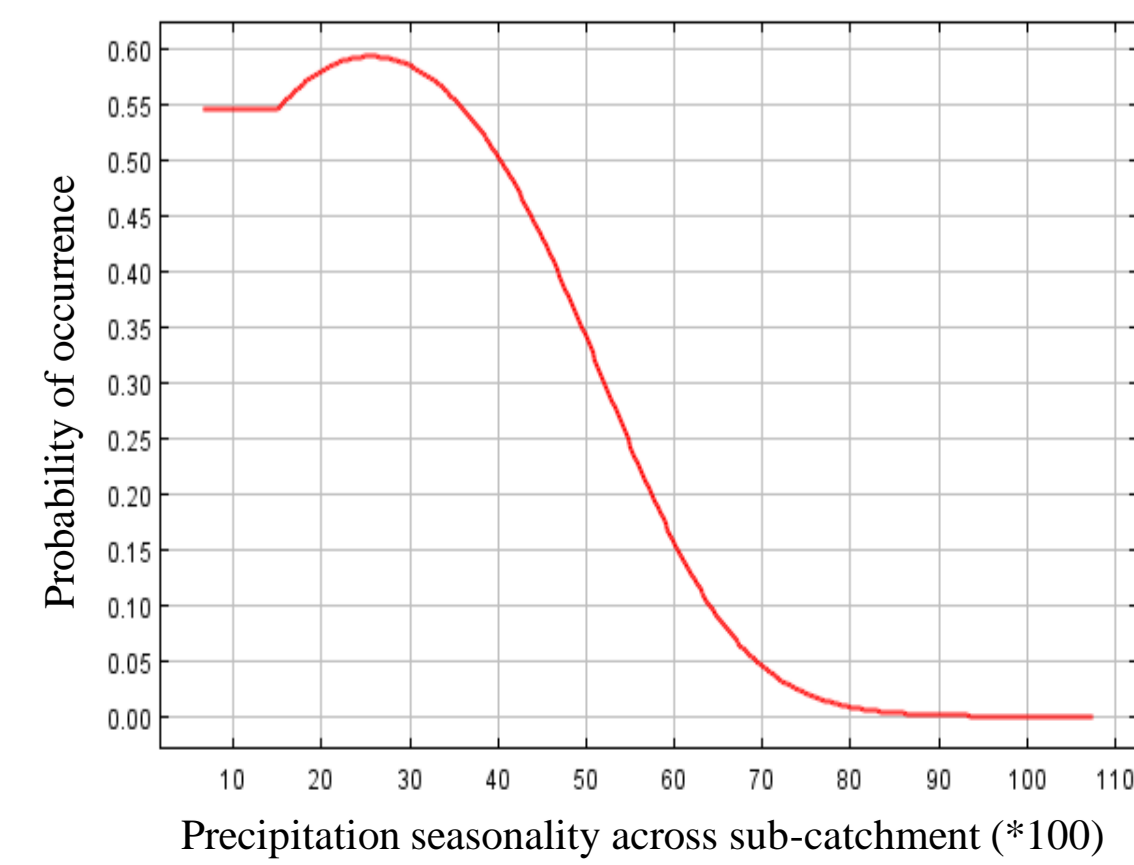

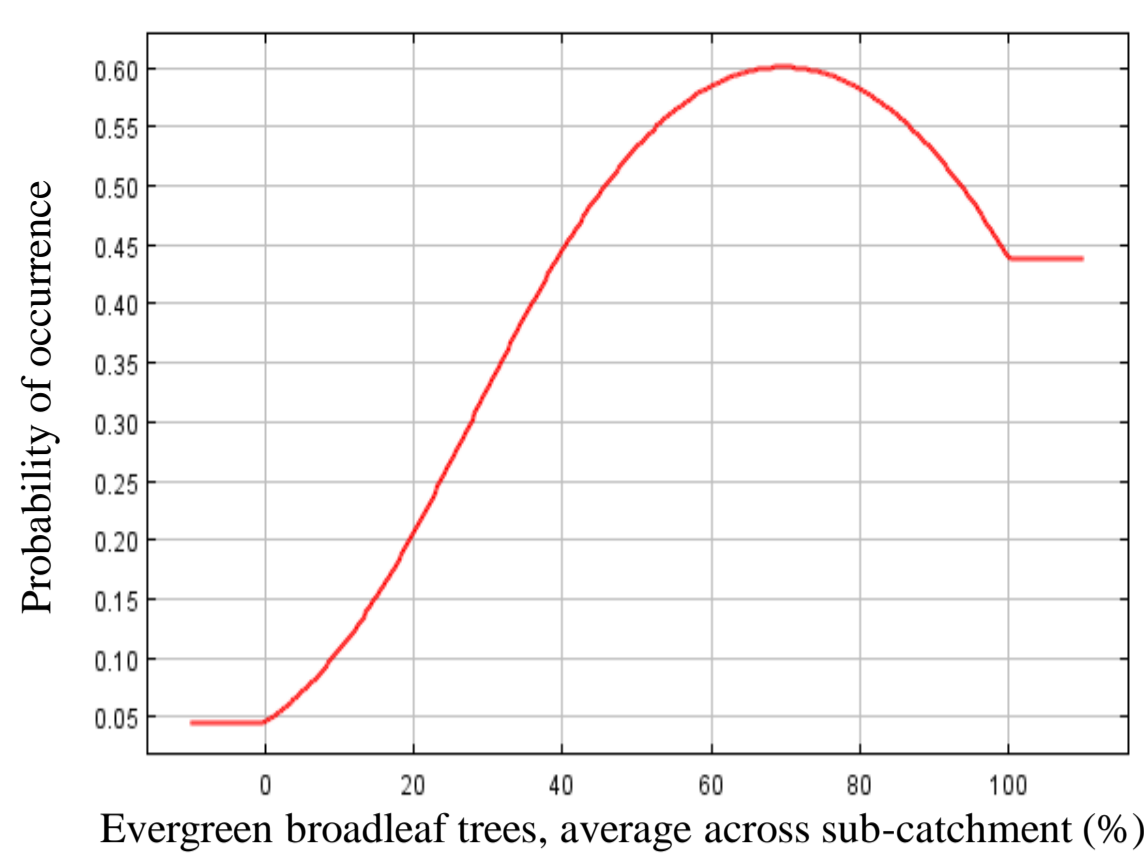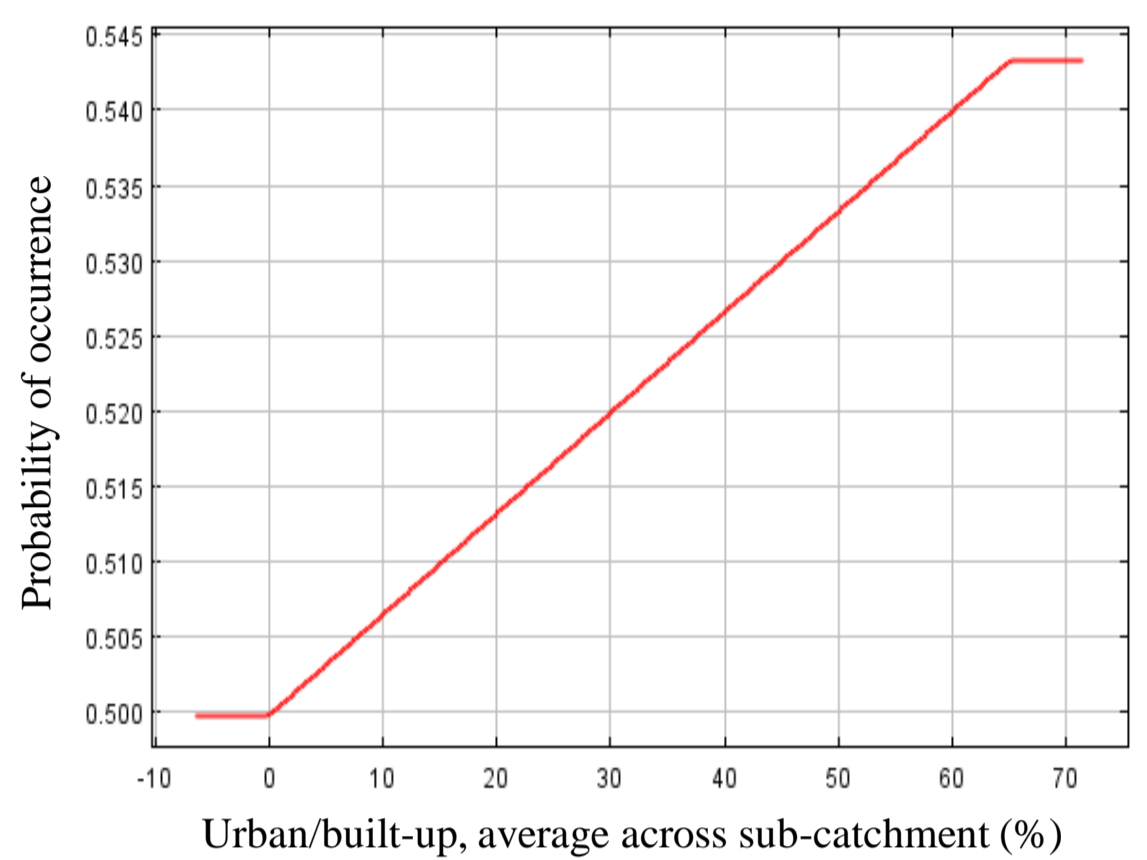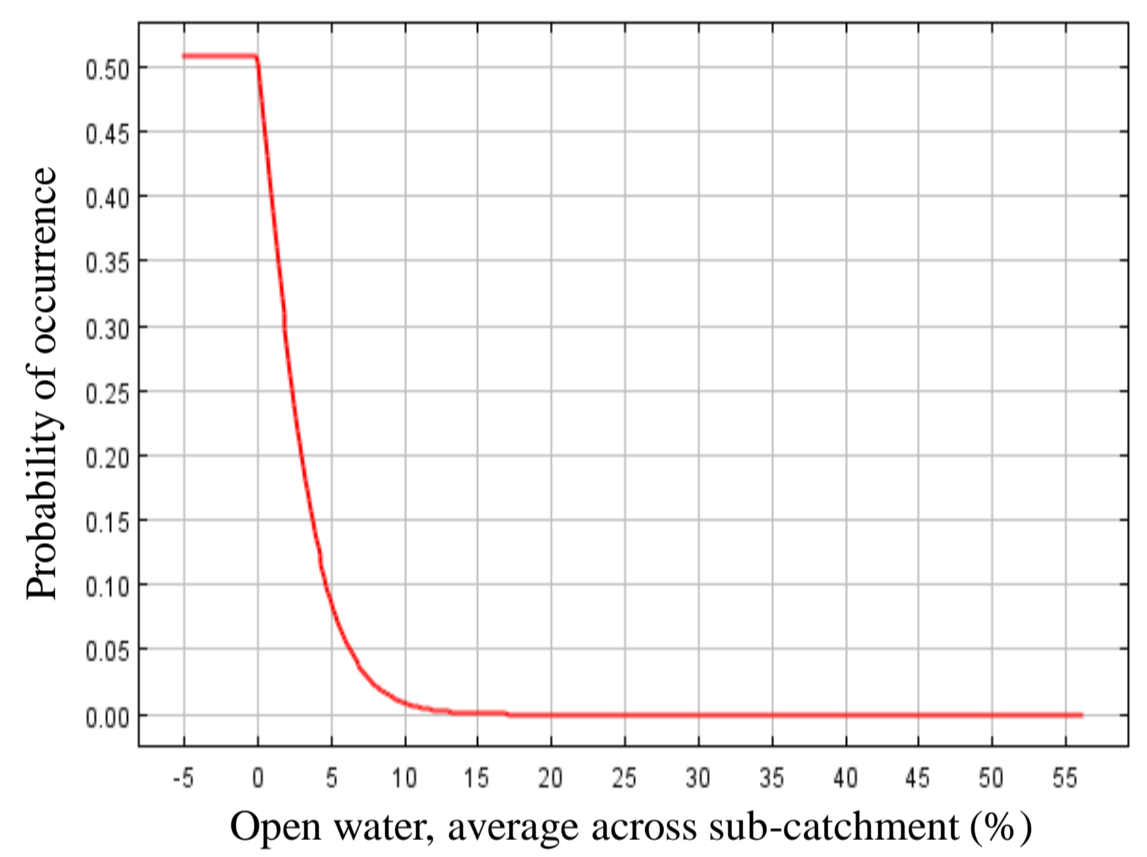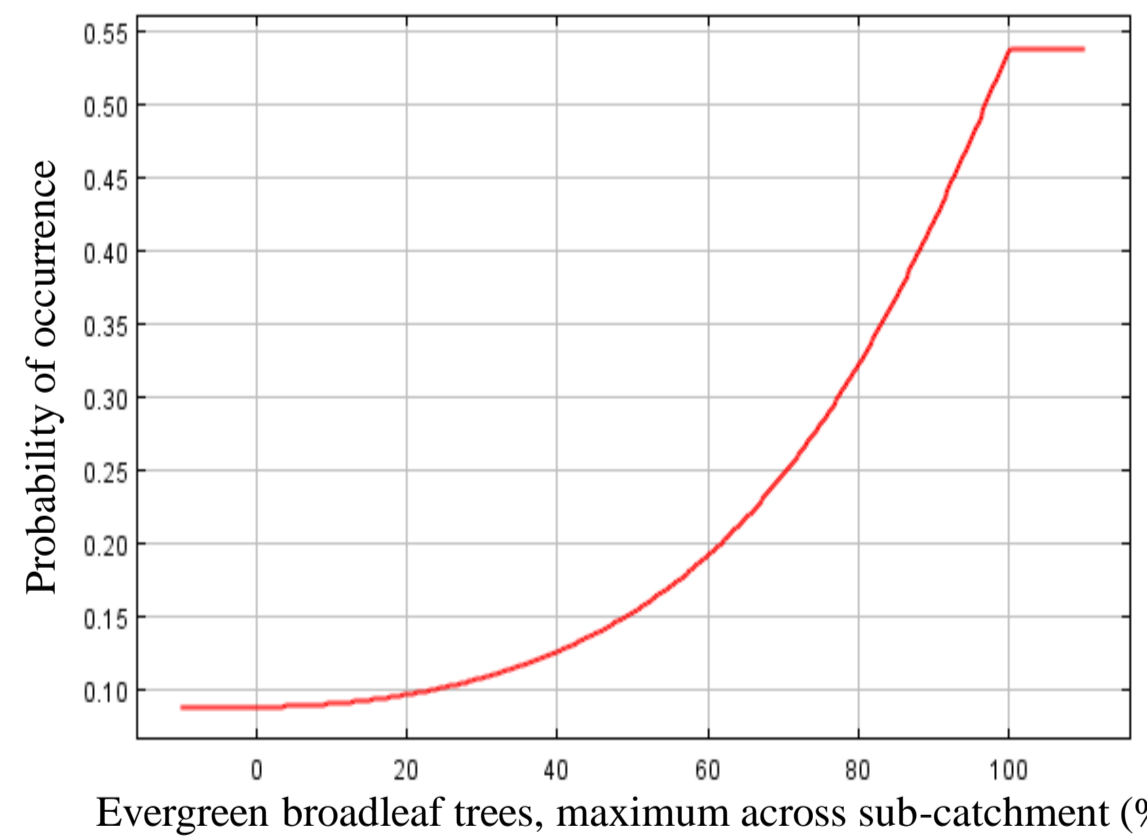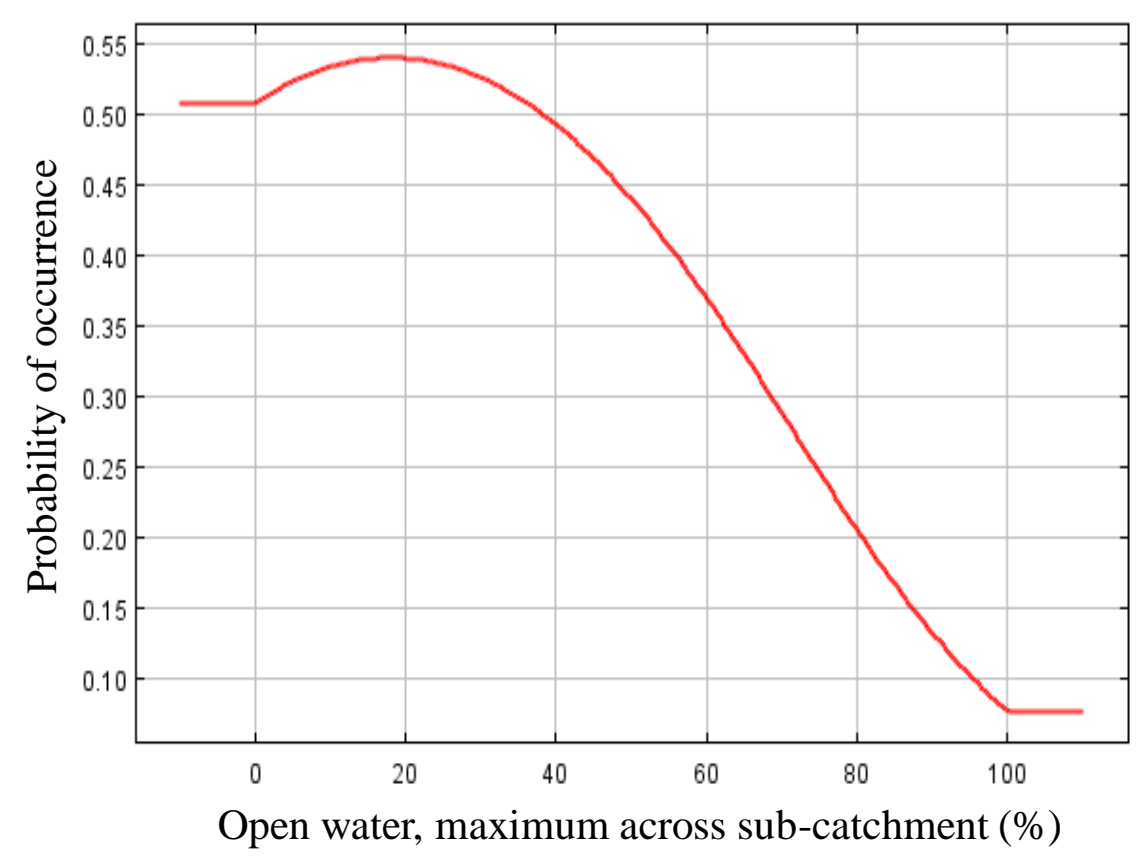

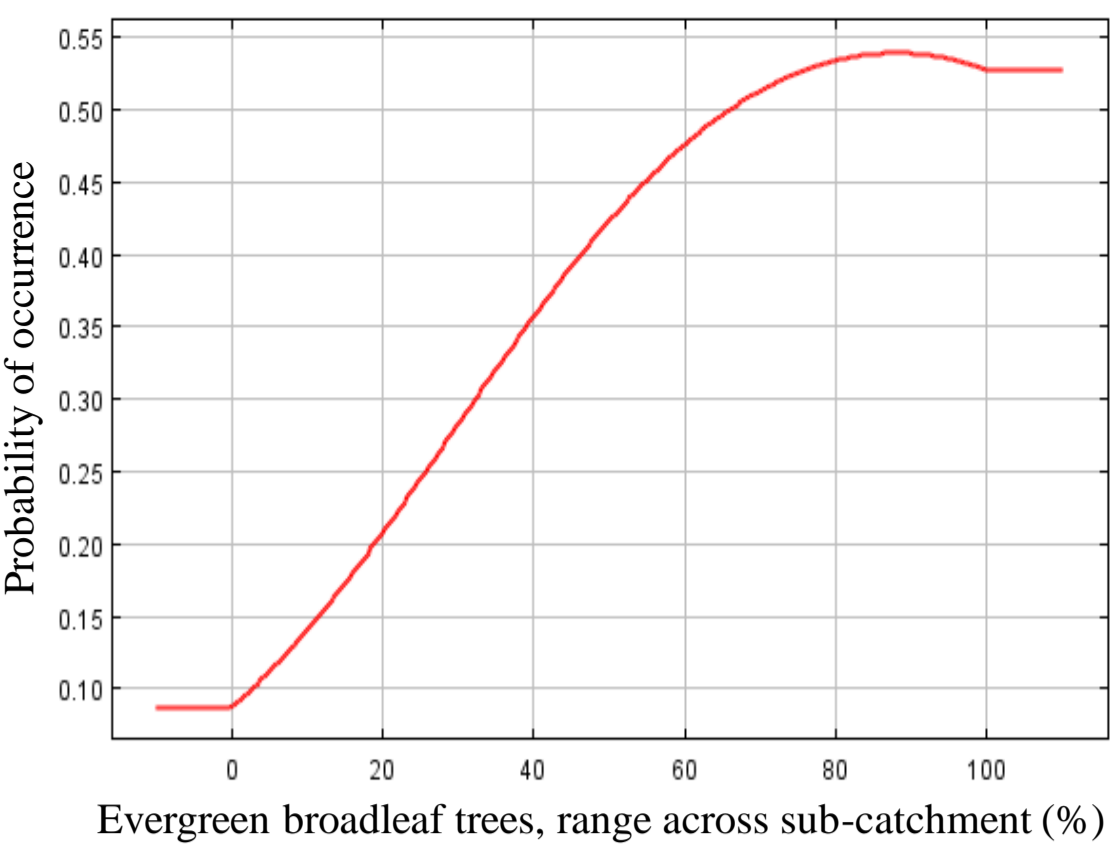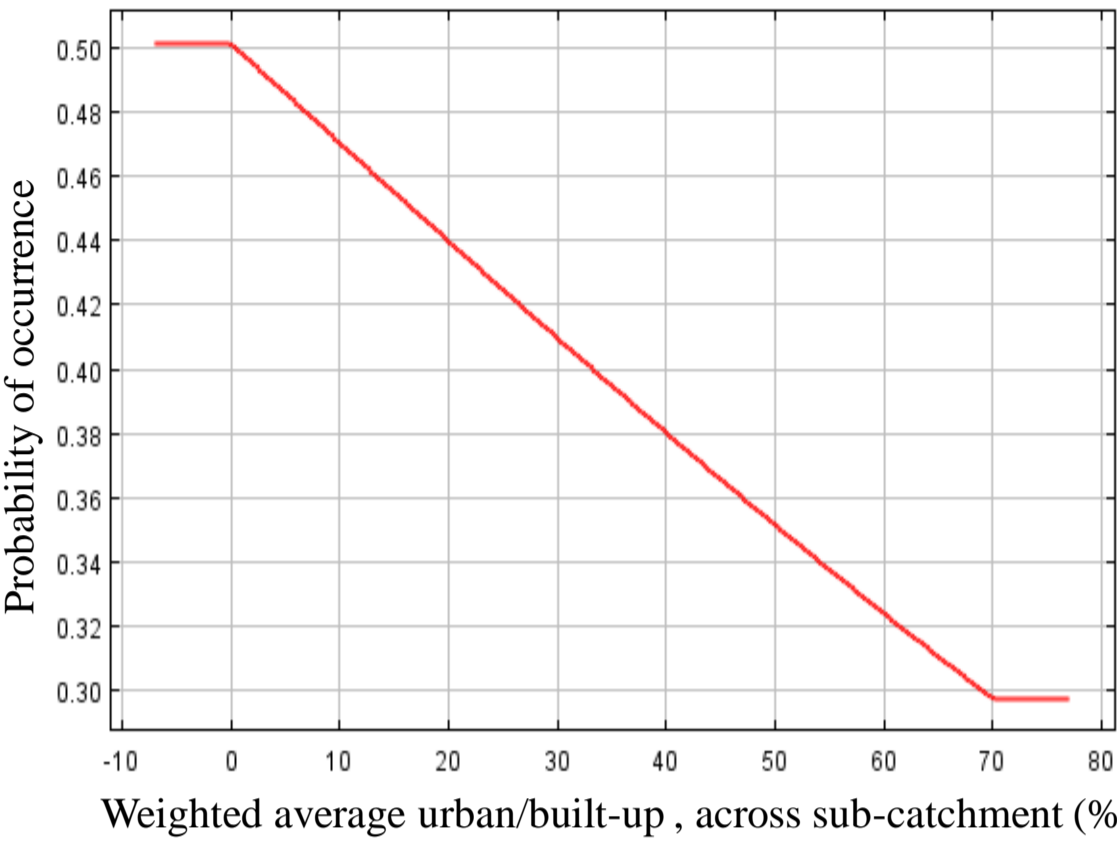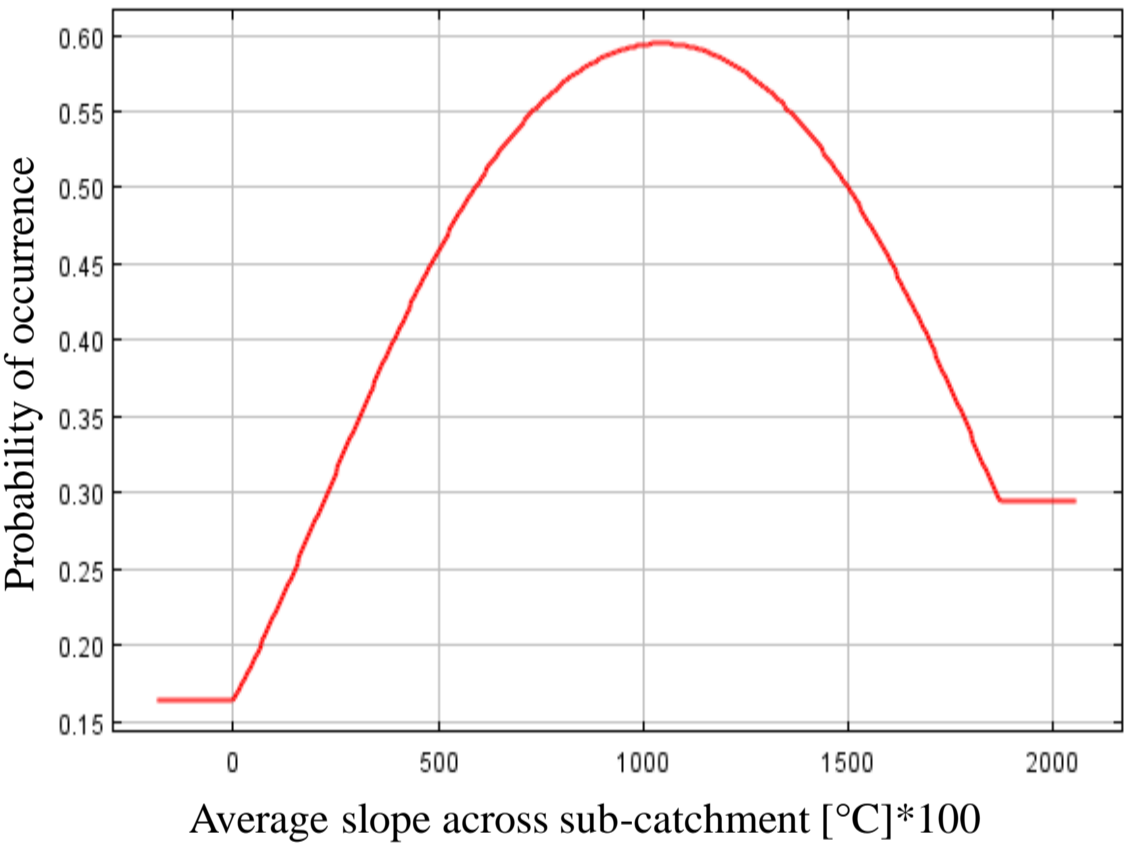

Supplement: S4 Fig — The first column represents the variables of the best model using both types of variables (on-site and upstream; model highlighted in bold in S3 Table) and in the second column, the response curves of the second best model using only on-site variables (see S3 Table). Response curves included in both models are presented side by side to facilitate comparison between them. (PDF) [file pone.0247876.s004.pdf]
