# Supplementary material for: Ecological niche modeling as an effective tool to predict the distribution of freshwater organisms: The case of the Sabaleta Brycon henni (Eigenmann, 1913)
Source: PLoS One. 2021 Mar 3;16(3):e0247876. doi: 10.1371/journal.pone.0247876 (PMC7928524; doi:10.1371/journal.pone.0247876)
Supplement: S1 Table — (PDF) [file pone.0247876.s005.pdf]

Database of occurrence sites of the Brycon henni species, as well as the data used to train and validate the model

Specie	Longitude	Latitude	Train or validation	Data antecedent	Presence/ absence
Brycon henni	-75.098087	6.787657	train	GBIF	1
Brycon henni	-75.139652	6.91468	train	GBIF	1
Brycon henni	-75.105287	6.781005	train	GBIF	1
Brycon henni	-75.144766	6.805534	train	GBIF	1
Brycon henni	-75.658361	5.110236	train	GBIF	1
Brycon henni	-75.071243	6.996357	train	GBIF	1
Brycon henni	-75.861844	6.4125	train	GBIF	1
Brycon henni	-75.137099	6.795467	train	GBIF	1
Brycon henni	-75.096002	7.12089	train	GBIF	1
Brycon henni	-75.711198	5.05999	train	GBIF	1
Brycon henni	-75.679107	5.129147	train	GBIF	1
Brycon henni	-75.19368	6.840182	train	GBIF	1
Brycon henni	-75.863335	5.088506	train	GBIF	1
Brycon henni	-75.704365	5.2625	train	GBIF	1
Brycon henni	-75.491669	5.443475	train	GBIF	1
Brycon henni	-75.938647	5.695833	train	GBIF	1
Brycon henni	-74.981967	7.320446	train	GBIF	1
Brycon henni	-75.234881	7.038065	train	GBIF	1
Brycon henni	-75.09416	7.354669	train	GBIF	1
Brycon henni	-75.954496	4.671149	train	GBIF	1
Brycon henni	-75.377326	5.761195	train	GBIF	1
Brycon henni	-76.537768	3.163131	train	GBIF	1
Brycon henni	-76.043557	3.904847	train	GBIF	1
Brycon henni	-75.928118	4.787793	train	GBIF	1
Brycon henni	-75.915278	4.7549	train	GBIF	1
Brycon henni	-75.928843	5.047125	train	GBIF	1
Brycon henni	-75.144901	6.37934	train	GBIF	1
Brycon henni	-75.252996	7.247597	train	GBIF	1
Brycon henni	-75.65156	4.546458	train	GBIF	1
Brycon henni	-75.703596	4.486486	train	GBIF	1
Brycon henni	-76.51062	3.023861	train	GBIF	1
Brycon henni	-75.963082	5.088275	train	GBIF	1
Brycon henni	-76.194341	4.071449	train	GBIF	1
Brycon henni	-75.145121	6.921516	train	GBIF	1
Brycon henni	-75.221555	7.14076	train	GBIF	1
Brycon henni	-75.248147	7.255979	train	GBIF	1
Brycon henni	-74.918773	6.404391	train	GBIF	1
Brycon henni	-74.912974	7.440313	train	GBIF	1
Brycon henni	-75.611409	4.940045	train	GBIF	1
Brycon henni	-75.619912	4.930623	train	GBIF	1
Brycon henni	-75.595556	4.627778	train	GBIF	1
Brycon henni	-75.702692	4.33139	train	GBIF	1

Brycon henni	-75.637434	4.988225	train	GBIF	1
Brycon henni	-75.661586	4.533	train	GBIF	1
Brycon henni	-75.687379	4.510608	train	GBIF	1
Brycon henni	-75.9941	5.1211	train	GBIF	1
Brycon henni	-76.68545	2.965789	train	GBIF	1
Brycon henni	-75.504552	7.006288	train	GBIF	1
Brycon henni	-75.657333	7.027917	train	GBIF	1
Brycon henni	-75.271097	6.223407	train	GBIF	1
Brycon henni	-75.164168	6.322054	train	GBIF	1
Brycon henni	-75.490222	5.436972	train	GBIF	1
Brycon henni	-75.7465	4.4243	train	GBIF	1
Brycon henni	-74.921959	6.279773	train	GBIF	1
Brycon henni	-75.6833	4.714	train	GBIF	1
Brycon henni	-75.831722	5.151778	train	GBIF	1
Brycon henni	-75.830028	5.145806	train	GBIF	1
Brycon henni	-75.793537	4.33267	train	GBIF	1
Brycon henni	-75.72556	4.406667	train	GBIF	1
Brycon henni	-75.800049	4.394047	train	GBIF	1
Brycon henni	-75.946667	5.080833	train	GBIF	1
Brycon henni	-75.178889	6.888917	train	GBIF	1
Brycon henni	-75.07775	6.307694	train	GBIF	1
Brycon henni	-75.17625	6.837833	train	GBIF	1
Brycon henni	-75.071307	6.308608	train	GBIF	1
Brycon henni	-75.17181	6.90614	train	GBIF	1
Brycon henni	-75.030806	6.272361	train	GBIF	1
Brycon henni	-74.99488	6.30714	train	GBIF	1
Brycon henni	-75.070306	6.300694	train	GBIF	1
Brycon henni	-75.181016	6.851559	train	GBIF	1
Brycon henni	-75.139444	6.930908	train	GBIF	1
Brycon henni	-75.138131	6.93723	train	GBIF	1
Brycon henni	-75.129928	6.945091	train	GBIF	1
Brycon henni	-75.920906	5.038224	train	GBIF	1
Brycon henni	-75.812755	4.240831	train	GBIF	1
Brycon henni	-76.554424	2.797608	train	GBIF	1
Brycon henni	-75.027898	7.125225	train	GBIF	1
Brycon henni	-75.637278	5.222806	train	GBIF	1
Brycon henni	-75.853452	4.713627	train	GBIF	1
Brycon henni	-75.846509	4.710955	train	GBIF	1
Brycon henni	-75.054776	6.369893	train	GBIF	1
Brycon henni	-75.861978	4.963737	train	GBIF	1
Brycon henni	-76.762331	2.805103	train	GBIF	1
Brycon henni	-75.88892	4.754417	train	GBIF	1
Brycon henni	-75.971551	5.96205	train	GBIF	1
Brycon henni	-75.69942	7.18436	train	GBIF	1
Brycon henni	-75.835534	6.397545	train	GBIF	1
Brycon henni	-76.770684	2.737496	train	GBIF	1
Brycon henni	-75.84345	6.87223	train	GBIF	1

Brycon henni	-75.411472	7.272097	train	GBIF	1
Brycon henni	-75.421359	7.254266	train	GBIF	1
Brycon henni	-75.895127	5.900044	train	GBIF	1
Brycon henni	-75.04568	6.430097	train	GBIF	1
Brycon henni	-75.856158	5.940483	train	GBIF	1
Brycon henni	-75.795375	6.542559	train	GBIF	1
Brycon henni	-75.619246	5.379056	train	GBIF	1
Brycon henni	-76.153482	4.188725	train	GBIF	1
Brycon henni	-75.677115	4.997248	train	GBIF	1
Brycon henni	-74.73455	5.206325	train	GBIF	1
Brycon henni	-75.672746	4.995215	train	GBIF	1
Brycon henni	-76.352665	3.882125	train	GBIF	1
Brycon henni	-75.847383	5.879428	train	GBIF	1
Brycon henni	-75.788179	4.704541	train	GBIF	1
Brycon henni	-75.748222	5.284139	train	GBIF	1
Brycon henni	-74.90459	7.44356	train	GBIF	1
Brycon henni	-76.411464	3.025594	train	GBIF	1
Brycon henni	-75.06046	7.18904	train	GBIF	1
Brycon henni	-74.77373	8.09689	train	GBIF	1
Brycon henni	-74.92811	6.49733	train	GBIF	1
Brycon henni	-76.523389	3.280432	train	GBIF	1
Brycon henni	-75.662757	5.699167	train	GBIF	1
Brycon henni	-74.981375	6.039689	train	GBIF	1
Brycon henni	-74.896309	6.512782	train	GBIF	1
Brycon henni	-74.941087	6.53027	train	GBIF	1
Brycon henni	-74.863906	6.503907	train	GBIF	1
Brycon henni	-76.512078	3.037325	train	GBIF	1
Brycon henni	-74.829103	6.495206	train	GBIF	1
Brycon henni	-76.511977	3.063934	train	GBIF	1
Brycon henni	-75.16825	5.909333	train	GBIF	1
Brycon henni	-74.872885	6.44449	train	GBIF	1
Brycon henni	-74.698253	6.382764	train	GBIF	1
Brycon henni	-76.503509	3.135606	train	GBIF	1
Brycon henni	-76.395768	3.446398	train	GBIF	1
Brycon henni	-74.640051	6.492554	train	GBIF	1
Brycon henni	-75.06873	7.29332	train	GBIF	1
Brycon henni	-75.045566	7.238007	train	GBIF	1
Brycon henni	-75.56107	5.44565	train	GBIF	1
Brycon henni	-76.495387	3.171273	train	GBIF	1
Brycon henni	-75.62862	5.220983	train	GBIF	1
Brycon henni	-75.488525	5.572071	train	GBIF	1
Brycon henni	-76.763845	2.737633	train	GBIF	1
Brycon henni	-75.514093	7.153405	train	GBIF	1
Brycon henni	-75.971677	4.771019	train	GBIF	1
Brycon henni	-75.085528	6.780056	train	GBIF	1
Brycon henni	-74.91821	6.50603	train	GBIF	1
Brycon henni	-75.142658	6.411279	train	GBIF	1

Brycon henni	-75.064629	7.303006	train	GBIF	1
Brycon henni	-75.854167	5.088806	train	GBIF	1
Brycon henni	-75.844987	4.688177	train	GBIF	1
Brycon henni	-75.971097	4.579553	train	GBIF	1
Brycon henni	-75.703524	4.436314	train	GBIF	1
Brycon henni	-75.862367	4.942345	train	GBIF	1
Brycon henni	-75.171646	6.897485	train	GBIF	1
Brycon henni	-75.9811	5.77309	train	GBIF	1
Brycon henni	-74.94693	6.490481	train	GBIF	1
Brycon henni	-75.615444	5.378222	train	GBIF	1
Brycon henni	-74.960712	6.496085	train	GBIF	1
Brycon henni	-75.120589	6.946323	train	GBIF	1
Brycon henni	-75.653194	5.459222	train	GBIF	1
Brycon henni	-75.556389	6.462778	train	GBIF	1
Brycon henni	-75.079944	6.770399	train	GBIF	1
Brycon henni	-75.969224	5.09677	train	GBIF	1
Brycon henni	-75.686318	7.171397	train	GBIF	1
Brycon henni	-75.763056	4.395834	train	GBIF	1
Brycon henni	-74.920618	7.271151	train	GBIF	1
Brycon henni	-75.787231	4.293183	train	GBIF	1
Brycon henni	-74.32917	10.9625	NA	NA	0
Brycon henni	-74.72917	9.270833	NA	NA	0
Brycon henni	-74.8875	8.054167	NA	NA	0
Brycon henni	-75.2375	7.754167	NA	NA	0
Brycon henni	-73.8875	7.1875	NA	NA	0
Brycon henni	-73.85417	7.170833	NA	NA	0
Brycon henni	-73.55417	7.1625	NA	NA	0
Brycon henni	-73.4125	7.104167	NA	NA	0
Brycon henni	-75.05417	7.029167	NA	NA	0
Brycon henni	-75.1125	6.770833	NA	NA	0
Brycon henni	-74.39583	6.504167	NA	NA	0
Brycon henni	-73.12083	6.495833	NA	NA	0
Brycon henni	-73.12083	6.4875	NA	NA	0
Brycon henni	-73.1125	6.479167	NA	NA	0
Brycon henni	-74.37083	6.345833	NA	NA	0
Brycon henni	-75.32083	6.245833	NA	NA	0
Brycon henni	-73.19583	6.104167	NA	NA	0
Brycon henni	-74.72917	5.7375	NA	NA	0
Brycon henni	-74.77917	5.695833	NA	NA	0
Brycon henni	-74.74583	5.6875	NA	NA	0
Brycon henni	-74.8625	5.670833	NA	NA	0
Brycon henni	-74.84583	5.670833	NA	NA	0
Brycon henni	-74.79583	5.670833	NA	NA	0
Brycon henni	-74.87083	5.6625	NA	NA	0
Brycon henni	-74.77083	5.6625	NA	NA	0
Brycon henni	-74.77917	5.654167	NA	NA	0
Brycon henni	-74.77083	5.654167	NA	NA	0

Brycon henni	-74.90417	5.620833	NA	NA	0
Brycon henni	-74.79583	5.6125	NA	NA	0
Brycon henni	-74.85417	5.579167	NA	NA	0
Brycon henni	-74.9375	5.570833	NA	NA	0
Brycon henni	-74.8625	5.570833	NA	NA	0
Brycon henni	-74.8875	5.5625	NA	NA	0
Brycon henni	-74.82917	5.320833	NA	NA	0
Brycon henni	-74.94583	5.3125	NA	NA	0
Brycon henni	-74.87917	5.295833	NA	NA	0
Brycon henni	-74.74583	5.2875	NA	NA	0
Brycon henni	-74.82917	5.254167	NA	NA	0
Brycon henni	-74.72917	5.204167	NA	NA	0
Brycon henni	-74.90417	4.3125	NA	NA	0
Brycon henni	-75.0875	4.295833	NA	NA	0
Brycon henni	-75.47083	2.620833	NA	NA	0
Brycon henni	-76.579806	2.779573	evaluation	Expeditions	NA
Brycon henni	-76.537455	2.83761	evaluation	Expeditions	NA
Brycon henni	-76.545233	2.878243	evaluation	Expeditions	NA
Brycon henni	-76.520961	2.978759	evaluation	Expeditions	NA
Brycon henni	-76.595635	3.244654	evaluation	Expeditions	NA
Brycon henni	-75.8558	4.372754	evaluation	Expeditions	NA
Brycon henni	-75.85529	4.71324	evaluation	Expeditions	NA
Brycon henni	-75.905286	4.945814	evaluation	Expeditions	NA
Brycon henni	-75.86213	4.962848	evaluation	Expeditions	NA
Brycon henni	-75.870026	5.096191	evaluation	Expeditions	NA
Brycon henni	-75.70424	5.26106	evaluation	Expeditions	NA
Brycon henni	-75.234667	7.034424	evaluation	Expeditions	NA
Brycon henni	-75.06873	7.29332	evaluation	Expeditions	NA
Brycon henni	-75.06982	7.30166	evaluation	Expeditions	NA
Brycon henni	-75.09068	7.35285	evaluation	Expeditions	NA
Brycon henni	-75.2429	7.22184	evaluation	Expeditions	NA
Brycon henni	-75.21513	7.13148	evaluation	Expeditions	NA
Brycon henni	-75.24785	7.25122	evaluation	Expeditions	NA
Brycon henni	-75.06046	7.18904	evaluation	Expeditions	NA
Brycon henni	-75.11577	7.10623	evaluation	Expeditions	NA
Brycon henni	-75.04069	7.24302	evaluation	Expeditions	NA
Brycon henni	76.04956	5.24926	evaluation	colleagues	NA
Brycon henni	-75.99226	5.07982	evaluation	colleagues	NA
Brycon henni	-75.88414	5.31558	evaluation	colleagues	NA
Brycon henni	-75.88441	5.30494	evaluation	colleagues	NA
Brycon henni	-75.87903	5.32965	evaluation	colleagues	NA
Brycon henni	-76.02756	4.94179	evaluation	colleagues	NA
Brycon henni	-76.0131	4.92373	evaluation	colleagues	NA
Brycon henni	-76.01779	4.93256	evaluation	colleagues	NA
Brycon henni	-75.99999	4.91887	evaluation	colleagues	NA
