# Supplementary material for: Ecological niche modeling as an effective tool to predict the distribution of freshwater organisms: The case of the Sabaleta Brycon henni (Eigenmann, 1913)
Source: PLoS One. 2021 Mar 3;16(3):e0247876. doi: 10.1371/journal.pone.0247876 (PMC7928524; doi:10.1371/journal.pone.0247876)
Supplement: S2 Table — The set of uncorrelated variables used to train the model are in bold type. (PDF) [file pone.0247876.s006.pdf]

For each single variable we provide the variable code, variable explanation, unit of measurement and native spatial grain

Code	Variable explanation	Unit	Source	Native spatial grain
bio_1	Annual Mean Temperature	[°C]*10	WorldClim	30 arcsec
**bio_2**	Mean Diurnal Range (Mean of monthly (max temp - min temp))	[°C]*10	WorldClim	30 arcsec
bio_3	Isothermality (BIO2/BIO7) (×100)	Dimensionless	WorldClim	30 arcsec
**bio_4**	Temperature Seasonality (standard deviation ×100)	[°C]*10	WorldClim	30 arcsec
bio_5	Max Temperature of Warmest Month	[°C]*10	WorldClim	30 arcsec
**bio_6**	Min Temperature of Coldest Month	[°C]*10	WorldClim	30 arcsec
bio_7	Temperature Annual Range (BIO5-BIO6)	[°C]*10	WorldClim	30 arcsec
bio_8	Mean Temperature of Wettest Quarter	[°C]*10	WorldClim	30 arcsec
bio_9	Mean Temperature of Driest Quarter	[°C]*10	WorldClim	30 arcsec
bio_10	Mean Temperature of Warmest Quarter	[°C]*10	WorldClim	30 arcsec
bio_11	Mean Temperature of Coldest Quarter	[°C]*10	WorldClim	30 arcsec
**bio_12**	Annual Precipitation	mm	WorldClim	30 arcsec
bio_13	Precipitation of Wettest Month	mm	WorldClim	30 arcsec
bio_14	Precipitation of Driest Month	mm	WorldClim	30 arcsec
**bio_15**	Precipitation Seasonality (Coefficient of Variation)	Dimensionless	WorldClim	30 arcsec
bio_16	Precipitation of Wettest Quarter	mm	WorldClim	30 arcsec
bio_17	Precipitation of Driest Quarter	mm	WorldClim	30 arcsec
bio_18	Precipitation of Warmest Quarter	mm	WorldClim	30 arcsec
bio_19	Precipitation of Coldest Quarter	mm	WorldClim	30 arcsec
havg_01	Bioclim 1, average across sub-catchment (water courses only)—see http://worldclim.org/bioclim	[°C] * 10	WorldClim	30 arcsec
**havg_02**	Bioclim 2, average across sub-catchment (water courses only)—see http://worldclim.org/bioclim	[°C] * 10	WorldClim	30 arcsec
**havg_03**	Bioclim 3, average across sub-catchment (water courses only)—see http://worldclim.org/bioclim	* 100	WorldClim	30 arcsec
**havg_04**	Bioclim 4, average across sub-catchment (water courses only)—see http://worldclim.org/bioclim	[°C] * 10	WorldClim	30 arcsec
havg_05	Bioclim 5, average across sub-catchment (water courses only)—see http://worldclim.org/bioclim	[°C] * 10	WorldClim	30 arcsec
**havg_06**	Bioclim 6, average across sub-catchment (water courses only)—see http://worldclim.org/bioclim	[°C] * 10	WorldClim	30 arcsec
havg_07	Bioclim 7, average across sub-catchment (water courses only)—see http://worldclim.org/bioclim	[°C] * 10	WorldClim	30 arcsec
havg_08	Bioclim 8, average across sub-catchment (water courses only)—see http://worldclim.org/bioclim	[°C] * 10	WorldClim	30 arcsec
havg_09	Bioclim 9, average across sub-catchment (water courses only)—see http://worldclim.org/bioclim	[°C] * 10	WorldClim	30 arcsec
havg_10	Bioclim 10, average across sub-catchment (water courses only)—see http://worldclim.org/bioclim	[°C] * 10	WorldClim	30 arcsec
havg_11	Bioclim 11, average across sub-catchment (water courses only)—see http://worldclim.org/bioclim	[°C] * 10	WorldClim	30 arcsec
havg_12	Bioclim 12, average across sub-catchment (water courses only)—see http://worldclim.org/bioclim	[mm]	WorldClim	30 arcsec
havg_13	Bioclim 13, average across sub-catchment (water courses only)—see http://worldclim.org/bioclim	[mm]	WorldClim	30 arcsec

havg_14	Bioclim 14, average across sub-catchment (water courses only)—see http://worldclim.org/bioclim	[mm]	WorldClim	30 arcsec
**havg_15**	Bioclim 15, average across sub-catchment (water courses only)—see http://worldclim.org/bioclim	* 100	WorldClim	30 arcsec
havg_16	Bioclim 16, average across sub-catchment (water courses only)—see http://worldclim.org/bioclim	[mm]	WorldClim	30 arcsec
havg_17	Bioclim 17, average across sub-catchment (water courses only)—see http://worldclim.org/bioclim	[mm]	WorldClim	30 arcsec
havg_18	Bioclim 18, average across sub-catchment (water courses only)—see http://worldclim.org/bioclim	[mm]	WorldClim	30 arcsec
havg_19	Bioclim 19, average across sub-catchment (water courses only)—see http://worldclim.org/bioclim	[mm]	WorldClim	30 arcsec
**lcmax_2**	Evergreen broadleaf trees, maximum across sub-catchment	[%]	Consensus land cover	30 arcsec
lcmax_7	Cultivated and managed vegetation, maximum across sub-catchment	[%]	Consensus land cover	30 arcsec
lcmax_9	Urban/built-up, maximum across sub-catchment	[%]	Consensus land cover	30 arcsec
**lcmax_12**	Open water, maximum across sub-catchment	[%]	Consensus land cover	30 arcsec
**lcran_2**	Evergreen broadleaf trees, range across sub-catchment	[%]	Consensus land cover	30 arcsec
lcran_7	Cultivated and managed vegetation, range across sub-catchment	[%]	Consensus land cover	30 arcsec
lcran_9	Urban/built-up, range across sub-catchment	[%]	Consensus land cover	30 arcsec
lcran_12	Open water, range across sub-catchment	[%]	Consensus land cover	30 arcsec
**lcavg_2**	Evergreen broadleaf trees, average across sub-catchment	[%]	Consensus land cover	30 arcsec
lcavg_7	Cultivated and managed vegetation, average across sub-catchment	[%]	Consensus land cover	30 arcsec
**lcavg_9**	Urban/built-up, average across sub-catchment	[%]	Consensus land cover	30 arcsec
**lcavg_12**	Open water, average across sub-catchment	[%]	Consensus land cover	30 arcsec
lcw_2	Weighted average land cover across sub-catchment: Evergreen broadleaf trees	[%]	Consensus land cover	30 arcsec
lcw_7	Weighted average land cover across sub-catchment: Cultivated and managed vegetation	[%]	Consensus land cover	30 arcsec
**lcw_9**	Weighted average land cover across sub-catchment: Urban/built-up	[%]	Consensus land cover	30 arcsec
lcw_12	Weighted average land cover across sub-catchment: Open water	[%]	Consensus land cover	30 arcsec
**Biolc_2**	Evergreen Broadleaf Trees	%	Consensus land cover	30 arcsec
**Biolc_9**	Urban/Built-up	%	Consensus land cover	30 arcsec
**Biolc_12**	Open Water	%	Consensus land cover	30 arcsec
**s_pto**	slope average	[°C]*100	HydroSHEDS	30 arcsec
**s_avg**	Average slope across sub-catchment	[°C]*100	HydroSHEDS	30 arcsec
**caudal**	Maximum, minimum and average flow from 1960 to 2000	[m3*s-1]	Barbarroja, FLO1K	30 arcsec
