# Supplementary material for: Ecological niche modeling as an effective tool to predict the distribution of freshwater organisms: The case of the Sabaleta Brycon henni (Eigenmann, 1913)
Source: PLoS One. 2021 Mar 3;16(3):e0247876. doi: 10.1371/journal.pone.0247876 (PMC7928524; doi:10.1371/journal.pone.0247876)
Supplement: S3 Table — We evaluated the performance of models with different values of the regularization parameter from 1 to 4, and lineal (l), quadratic (q), product (p), threshold (t), and hinge (h) relationships. The number of free parameters (k); rescaled Akaike information criterion (ΔAICc); Akaike weights (w.AIC) and the area under the curve (AUC), which is a measure of the model’s performance in relation to a null model. Best model was chosen based on its AICc and its AUC value. In bold type the best overall model is highlighted and models are organized by the types of predictor variables used (on-site or on-site and upstream) and AICc (lowest to highest). (PDF) [file pone.0247876.s007.pdf]

Brycon henni distribution models using different sets of aquatic predictors (on-site or on-site and upstream)

Model	regularization parameter	l	q	p	t	h	*k*	AIC_c	ΔAIC_c	w.AIC	AUC
on-site	4	x	x	x	x	x	20	2979.85	18.30	1.5E-05	0.845
on-site	1	x	x				14	2995.44	19.58	4.1E-04	0.841
on-site	4	x	x	x	x		18	3000.44	20.59	3.4E-05	0.816
on-site	3	x	x	x	x	x	37	3011.55	31.69	1.3E-07	0.863
on-site	3	x	x	x	x		32	3013.16	33.31	5.9E-08	0.839
on-site	2	x	x				11	3017.05	37.20	8.4E-09	0.819
on-site	2	x	x	x	x	x	47	3017.70	37.85	6.0E-09	0.880
on-site	4	x	x	x			24	3018.45	38.59	4.2E-09	0.785
on-site	3	x	x				11	3037.19	57.34	3.5E-13	0.794
on-site	2	x	x	x	x		48	3042.29	62.43	2.8E-14	0.874
on-site	4	x	x				11	3057.07	77.22	1.7E-17	0.769
on-site	3	x	x	x			46	3064.89	85.03	3.4E-19	0.805
on-site	2	x	x	x			57	3083.00	103.15	4.0E-23	0.833
on-site	1	x	x	x	x	x	69	3089.15	109.30	1.8E-24	0.895
on-site	1	x					9	3137.89	158.03	4.8E-35	0.761
on-site	2	x					9	3138.65	158.80	3.3E-35	0.762
on-site	3	x					9	3139.77	159.92	1.9E-35	0.763
on-site	4	x					9	3141.21	161.35	9.2E-36	0.764
on-site	1	x	x	x	x		77	3162.14	182.29	2.6E-40	0.896
on-site	1	x	x	x			80	3185.14	205.29	2.6E-45	0.847
**on-site+Upstream**	**1**	x	x				**30**	**2881.08**	**0**	**0.9999**	**0.899**
on-site+Upstream	2	x	x				27	2904.67	23.59	7.6E-06	0.882
on-site+Upstream	4	x	x	x	x		41	2926.72	45.63	1.2E-10	0.881
on-site+Upstream	3	x	x				26	2934.71	53.62	2.3E-12	0.855
on-site+Upstream	4	x	x				21	2944.78	63.70	1.5E-14	0.847
on-site+Upstream	4	x	x	x	x	x	54	2948.16	67.07	2.7E-15	0.891
on-site+Upstream	3	x	x	x	x		55	2955.50	74.42	6.9E-17	0.887
on-site+Upstream	3	x	x	x	x	x	63	2970.41	89.32	4.0E-20	0.897
on-site+Upstream	3	x	x	x			59	2992.38	111.30	6.8E-25	0.871
on-site+Upstream	4	x	x	x			51	2997.09	116.00	6.5E-26	0.861
on-site+Upstream	1	x					19	3026.79	145.71	2.3E-32	0.841
on-site+Upstream	2	x					18	3027.56	146.47	1.6E-32	0.837
on-site+Upstream	4	x					15	3030.80	149.72	3.1E-33	0.826
on-site+Upstream	3	x					18	3032.50	151.42	1.3E-33	0.833
on-site+Upstream	2	x	x	x	x		75	3038.45	157.36	6.8E-35	0.903
on-site+Upstream	2	x	x	x	x	x	87	3118.27	237.18	3.1E-52	0.906
on-site+Upstream	2	x	x	x			88	3165.12	284.03	2.1E-62	0.887
on-site+Upstream	1	x	x	x	x		109	3428.62	547.54	1.3E-119	0.920
on-site+Upstream	1	x	x	x			113	3526.44	645.35	7.3E-141	0.894
on-site+Upstream	1	x	x	x	x	x	126	3992.93	1111.84	3.7E-242	0.920
