# Supplementary material for: Ecological niche modeling as an effective tool to predict the distribution of freshwater organisms: The case of the Sabaleta Brycon henni (Eigenmann, 1913)
Source: PLoS One. 2021 Mar 3;16(3):e0247876. doi: 10.1371/journal.pone.0247876 (PMC7928524; doi:10.1371/journal.pone.0247876)
Supplement: Attachment — Submitted filename: Response to Reviewers.docx [file pone.0247876.s009.docx]

PONE-D-20-23545
Ecological niche modeling as an effective tool to predict the distribution of freshwater organisms: the case of the Sabaleta *Brycon henni* (Eigenmann, 1913**)**

February 12, 2021

**Pedro Abellán**
Academic Editor
PLOS ONE

Dear Editor,

On behalf of my coauthors, I would like to thank you and the reviewers for the constructive criticism. We have addressed all comments and hope this version of the manuscript meets your expectations.

Response to each comment is below in blue.

**Editor’s Comments**

The previous reviewer and myself have reviewed the new version of the manuscript, and we both agree that the paper is much improved, and that the authors have addressed the previous comments seriously and effectively. The reviewer has recommended publication, but also suggests some minor yet important revisions to your manuscript that should be addressed. Therefore, I invite you to respond to the reviewer' comments and revise your manuscript. Overall, the reviewer has pointed the need of discuss the relative importance of the variables, and the potential confounding use of the word “connectivity” when referring to upstream or watershed factors (note that stream connectivity usually refers to the longitudinal connections or pathways along the length of a stream).

Response: The second and third paragraph of the discussion, in addition to Table 1, include all information about the relative importance of variables, and whether they can be interpreted as complementary or redundant. The most important variables in the model included annual precipitacion, precipitation seasonality and minimum temperature – both on site and upstream -, and percent open water. We included a sentence in the third paragraph that describes how these variables relate to relative species’ occurrence rate: “Response curves of these variables indicate that the Sabaleta can be found in sites with relatively high annual precipitation (> 2500 mm), little percent open water (<10 %), profiles of minimum temperatures that include both low and high elevation sites (~10-20 C), and relatively little precipitation seasonality (S4 Fig).”

We also edited the use of the word "Connectivity" in the last paragraph of the discussion. Our intention was to emphasize the importance of including variables at multiple scales (on site and upstream) in ENM of freshwater species.

**Comments to the Author**

**Reviewer #1:** The paper has been improved considerably. However, I would like to see some text on the "relative" importance of specific variables on the distribution of the target species (as they come from a pool of higly correlated variables). In the same way, I also think that the concept of "conectivity" is mixed in the last paragraph of the discusion. The authors are mixing the imporance of conectivity among sites for the conservation of freshwater fauna and the importance of variables that operate at basin scales to predict the species occurence.

Response: Thank you, we rephrased these sentences to avoid confusion.

Thanks again for all the support. Please let us know of any additional requirement. Looking forward to hear from you. Best,

Daniel Valencia
